# Spin-Polarized Nonferromagnetic Surfaces for Electrocatalysis: Chemo-Spintronics

**DOI:** 10.1021/jacs.5c16824

**Published:** 2025-12-19

**Authors:** Hansaem Jang, Daniel Roe, Harry E. Taylor, Emiliano Poli, Alex S. Walton, Gilberto Teobaldi, Oscar Cespedes, Alexander J. Cowan

**Affiliations:** † Department of Chemistry and Stephenson Institute for Renewable Energy, 4591 University of Liverpool, Liverpool L69 7ZF, U.K.; ‡ School of Physics and Astronomy, 4468 University of Leeds, Leeds LS2 9JT, U.K.; § Department of Chemistry and Photon Science Institute, 5292 The University of Manchester, Manchester M13 9PL, U.K.; ∥ Scientific Computing Department, Science & Technology Facilities Council UKRI, Rutherford Appleton Laboratory, Didcot OX11 0QX, U.K.

## Abstract

Catalysts achieve changes in the rate through modification of the free energy of adsorbed intermediates and transition states (TrS). Binding energies of intermediates and TrS are strongly correlated, and modifications in catalyst composition are often ineffective in breaking these correlations, leading to minimal change in rate. Such scaling relationships are reported throughout catalysis. The surface spin state of a magnetic metal can change adsorption energies, offering a way to overcome scaling relationships. However, experimentally, this approach appears reliant on the use of ferromagnetic materials, limiting applicability. Here, we show that tunable changes in electrocatalytic activity for the hydrogen evolution reaction (HER) can be achieved at (originally) nonmagnetic metals (Au and Pt) through the use of a multilayer electrode structure that contains a ferromagnetic alloy (CoB) beneath a thin (5–20 nm) film of Pt or Au. Analysis of the dependence of the catalytic current on the thickness of the Au or Pt capping layer and on the direction of the stray magnetic field allows us to rule out the presence of magnetohydrodynamic effects. Instead, we conclude that transfer of ferromagnetism from the ferromagnet to the Au or Pt takes place through proximity-induced magnetism (PIM) via exchange interactions and/or a spin polarized current. Density Functional Theory simulations trace changes in the breaking of the scaling relationship for the Tafel HER mechanism. Overall, our experiments show that thin-film electrodes, based on routine structures from the spintronics community, are a potentially versatile platform for achieving spin-polarized catalysis at originally nonmagnetic metals.

## Graphical abstract

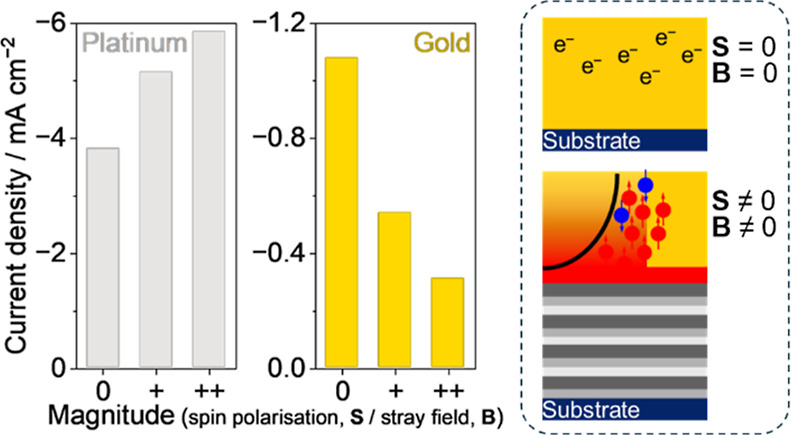

## 1 Introduction

Scaling relationships (i.e., linear correlations between physicochemical properties such as adsorption energies of reaction intermediates, allowing one to be governed by or predicted from another) exist across catalysis as the binding energies of surface intermediates are typically interrelated. Thus, optimization of the catalyst structure to achieve a change in the binding energy of one intermediate will lead to a change in the binding energy of the other species along the reaction pathway. Such effects limit the degrees of freedom available within catalyst design, often placing an apparent upper limit in achievable catalytic activity, which is often visualized in the form of the “peak of the volcano” in a 2D catalytic activity–descriptor plot.^1^ Recently, Cao and Norskov showed through Density Functional Theory (DFT) calculations that the adsorption energy of a surface species on a metal is strongly affected by spin-polarization of the surface.^2^ It was noted that spin effects differed depending on the nature of the adsorbate and on the presence of additional adsorbates, offering a potential route to break scaling relationships. These calculations may in-part at least offer a rationale of recent experimental results relating to reports of increased rates for the electrocatalytic oxygen evolution reaction at ferromagnetic electrodes^3–7^ and for a wider range of electrocatalytic reactions.^8^ However, experimentally evidencing surface-spin polarization control during (electro)­catalysis is complicated with the magnitude and source of the effect debated.^9^ A common complication is hydrodynamic effects caused by the presence of stray magnetic fields that some have proposed may be the dominant cause of the change in electrocatalytic rates.^10–12^ Although the recent studies have contributed to the understanding of the profound effects of magnetic fields on electron spins in materials with different magnetic properties (e.g., diamagnetic, ferromagnetic, ferrimagnetic, antiferromagnetic, paramagnetic, and superparamagnetic) as well as magnetic gradients,^13,14^ tuning of surface spin-polarization is also limited to the choice of the ferromagnetic material and hard to control experimentally in the presence of coadsorbates.

Demonstrating a straightforward route to extend spin-control effects beyond ferromagnetic metals would open a new route to the scalable design of new catalytic surfaces with profoundly different activities using the existing suite of known chemical compositions. It may also open for exploration of new, not naturally accessible, chemistries and catalytic routes based on standard, readily available transition metals. Exchange interactions in proximity-induced magnetism (i.e., a phenomenon where a nonmagnetic material obtains magnetic properties when placed near a magnetic material) and charge currents from ferromagnetic electrodes can be used to induce spin order and spin polarization in conventionally nonmagnetic heavy metals with large spin–orbit coupling (SOC).^15–18^ With this in mind, we hypothesized that multilayer samples containing ferromagnetic alloy (CoB) beneath a standard, nonmagnetic (NM), heavy transition-metal thin-film electrode would provide a versatile platform for achieving spin-polarized catalysts (Figure 1). One very recent study has examined a Co/Pt multilayer structure for electrocatalytic ammonia oxidation and identified a change in catalytic current assigned to spin-polarization of surface species on the perpendicular magnetic anisotropy (PMA) structure.^19^ However, the wider applicability of the approach to achieve spin-polarization catalysis to other nonferromagnetic metals has not been explored. Also unexplored is that control of the thickness of the ferromagnetic layers, which can enable the generation of samples with either PMA (i.e., a stray magnetic field will be present at the interface with the electrolyte with the magnetic field gradient controlled by the density of out-of-plane domains) or with in-plane (IP) magnetization (i.e., minimal stray field and no magnetic field gradient will be present at the interface with the electrode)—see Table 1. Both PMA and IP samples will generate proximity effects and inject spin polarized currents in the cap layer providing a way to discriminate between spin-polarization and magnetohydrodynamic effects during catalysis. Here, we have prepared a series of proximitized Au and Pt catalytic surfaces for use in electrocatalysis with control of the stray magnetic field to provide unambiguous evidence for spin-polarized catalysis at nonmagnetic materials.

**Figure 1:**
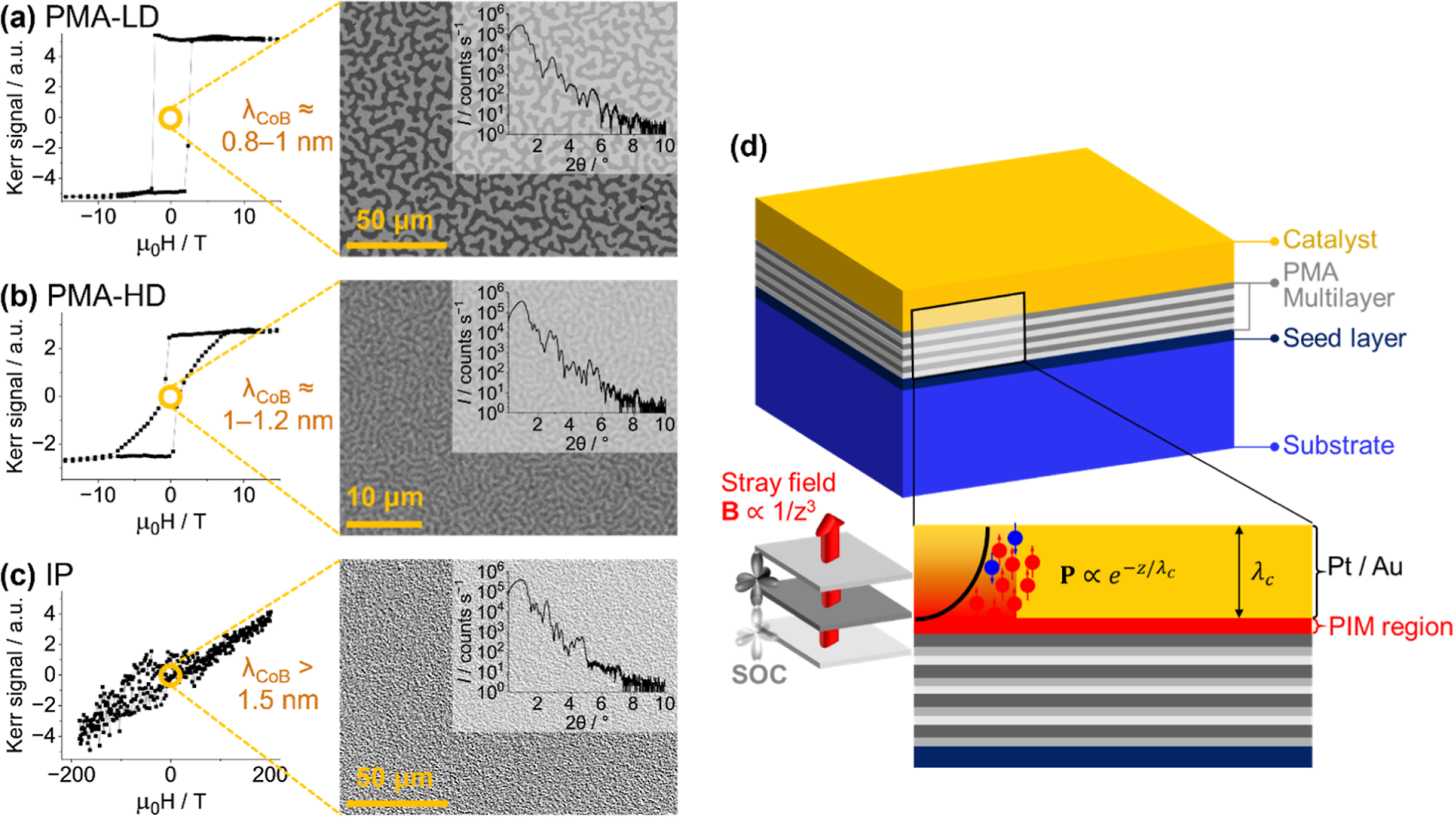
Left (a–c): Magneto-optic Kerr effect microscopy measured with an out-of-plane applied field and X-ray reflectivity characterization of the proximitized magnetic multilayers. Right (d): Schematic of the samples. The direction of the magnetization goes from being in-plane (IP (c)) to out-of-plane due to the perpendicular magnetic anisotropy (PMA (a,b)) induced by SOC at the interface with heavy metals when the CoB layer (i.e., the middle layer of each trilayer in the multilayer stack) thickness is below some 1.2 nm—note the change in scale for the magnetic field for IP samples. The density of maze domains can be tuned from low density (LD) and high density (HD) by adjusting either the number of trilayer repeats in the multilayer structure or the thickness of the CoB layer (λ_CoB_). PMA samples with HD domains will generate a larger magnetic field gradient and therefore may affect the ionic diffusion more strongly and are expected to have a greater impact on hydrodynamics. Note: in this work, no external magnetic field is applied but there exists an intrinsic stray field generated by the magnetic film either due to the PMA or granular structure.

**Table 1:** Variation of Electrode Surface Properties for Samples with Magnetic Structures with PMA (HD and LD Domains) or IP Magnetism as the Electrocatalyst Cap Layer Thickness (λ_c_) Is Changed (Relative to Spin Diffusion Length (λ_sd_))^a^

surface property	sample (λ_c_ vs. λ_sd_)		
	λ_c_ < λ_sd_ / Au (5, 10 nm)	λ_c_ ≈ λ_sd_ / Pt (5 nm), Au (20 nm)	λ_c_ > λ_sd_ / Pt (10 nm)
spin polarization (**P**)	high	moderate	low
magnetic order	yes	no	no
magnetic field (**H**)	PMA—strong	PMA—strong	PMA—medium
	IP—weak	IP—negligible	IP—nil
magnetic field gradient (∇**H**)	PMA (HD)—very strong	PMA (HD)—very strong	PMA (HD)—strong
	PMA (LD)—strong	PMA (LD)—strong	PMA (LD)—strong
	IP—nil	IP—nil	IP—nil

a Spin diffusion length is the mean distance over which spin-polarized electrons can travel in a material without losing their spin orientation. At 300 K, Au and Pt typically exhibit spin diffusion lengths of over 30 nm and less than 3 nm, respectively—for details, see Results and Discussion. Recognizing the ratio of λ_sd_ to λ_c_ is important, as it can provide insights into whether magnetic moments can be sustained at the surface.

## 2 Methods

### 2.1 Electrode Preparation

Working electrodes were prepared on ⟨100⟩ thermally oxidized silicon substrate (Si-Mat, Germany) cut to a square (10 mm × 10 mm). Substrates were cleaned in acetone and then isopropanol before they were loaded into the vacuum chamber for growth. Samples were deposited using DC magnetron sputtering in an Ar pressure of ca. 3 × 10^–3^ mbar within a vacuum chamber with a base pressure of the order of 10^–8^ mbar. An alloy target of composition Co_68_B_32_ is used to grow the CoB layers and all other layers are deposited from 99.99% pure single-element targets. It is worth noting that the CoB composition in the structures may deviate from 68:32, as the target composition can vary over time due to the different sputtering rates of cobalt and boron. Nevertheless, the primary role of boron in CoB alloys for spintronics is to reduce grain size and create smooth interfaces that favor perpendicular magnetic anisotropy (PMA) in ultrathin films when interfaced with a heavy metal such as Pt. Generation of PMA is confirmed in Figure 1. The catalyst is also grown in the same chamber. Magnetic superlattices were grown in-plane (IP; thicker CoB layers) or PMA (thinner CoB layers) to study the effect of stray fields present in PMA samples. Larger or smaller out-of-plane domains in PMA multilayers were obtained by fine-tuning the metal layer thicknesses in order to analyze the effect of magnetic field gradients (Figure 1). The domain state was set on all samples before catalysis measurements by applying an AC magnetic field of 20 mT which is gradually reduced to 0 mT. Magneto-optic Kerr effect microscopy and X-ray reflectivity measurements were used to characterize samples prior to electrochemical study.

### 2.2 Electrolyte Preparation

A 0.5 M KHCO_3_ electrolyte was prepared by dissolving the chemical (≥99.5%; Sigma-Aldrich, Spain) in deionized water, after which 1 L of the solution was electrochemically purified to remove trace-level impurities at a constant current of 0.1 mA for 16 h under agitation by magnetic stirring without purging. This process employed a two-electrode system where the negative and positive electrodes are a titanium plate (2 cm × 1 cm) and a platinum coil (16 cm), respectively. The purified electrolyte was purged in Ar (99.998%; BOC, United Kingdom) at a flow rate of 20 cm^3^ min^–1^ (sccm) for 30 min and then the basicity was measured as pH 8.8 using a pH probe.

### 2.3 Electroanalysis Preparation: Pt

A Pt-capped sample was used as the working electrode, with an exposed geometric surface area of 0.09 cm^2^ (0.3 × 0.3 cm), ensuring that neither the edges of the capping layer nor the substrate were exposed. A Ag/AgCl electrode (3 M KCl, +210 mV versus SHE; Redoxme, Sweden), equipped with a ceramic frit fused into the glass body, was used as the reference electrode. A Pt coil (25 cm), flame-cleaned prior to use, was employed as the counter electrode. A H-type cell (R-A-ECSYNTH_E/S, Redoxme, Sweden) was used as the electrochemical cell. The working and reference electrodes were placed in the working electrode chamber, while the counter electrode was placed in the counter electrode chamber. The two chambers were separated by a membrane (Fumasep FS-990-PK, Fumatech, Germany). The membrane was soaked in deionized water overnight prior to use. For electrochemical measurements, both chambers were filled with purified electrolyte. The catholyte was continuously purged with Ar at a flow rate of 20 sccm, starting at least 30 min prior to the measurements. Care was taken to position the Ar gas inlet sufficiently far from the catalyst surface, ensuring Ar bubbles were not directly blown onto or reached the surface. All tests were conducted without stirrer bars. Prior to electrochemical analysis, sample surfaces underwent cathodic treatment; for this, multiple linear sweep voltammetry (LSV) scans were performed from 0.1 to −0.15 V versus reversible hydrogen electrode (RHE) at a scan rate of 5 mV s^–1^ until the polarization curve exhibited a monotonic decrease over the scan. Thereafter, the sample was rinsed with deionized water, dried thoroughly using N_2_, and reassembled for electrocatalysis.

### 2.4 Electroanalysis Preparation: Au

A Au-capped sample was used as the working electrode, with an exposed geometric surface area of 0.5 cm^2^ (0.7979 cm diameter), ensuring that neither the edges of the capping layer nor the substrate were exposed. A Ag/AgCl electrode (3 M KCl, +210 mV versus SHE; Redoxme, Sweden), equipped with a ceramic frit fused into the glass body, was used as the reference electrode. A Pt coil (25 cm), flame-cleaned prior to use, was employed as the counter electrode. A H-type cell (C-A-BM_FC_HC-50-10x10, Redoxme, Sweden) was used as the electrochemical cell. The working and reference electrodes were placed in the working electrode chamber, while the counter electrode was placed in the counter electrode chamber. The two chambers were separated by a membrane (Fumasep FS-990-PK, Fumatech, Germany). The membrane was soaked in deionized water overnight prior to use. For electrochemical measurements, both chambers were filled with purified electrolyte. The catholyte was continuously purged with Ar at a flow rate of 20 sccm, starting at least 30 min prior to the measurements. Care was taken to position the Ar gas inlet sufficiently far from the catalyst surface, ensuring Ar bubbles were not directly blown onto or reached the surface. All tests were conducted without stirrer bars. Prior to electrochemical analysis, sample surfaces underwent cathodic treatment; for this, 19 LSV scans were performed from 0.1 to −0.6 V versus RHE at a scan rate of 20 mV s^–1^. This pretreatment process differs from that of the Pt samples, as unlike Pt, Au exhibits significant overpotentials for hydrogen evolution or oxidation reactions, thereby providing a wider non-Faradaic potential window.

### 2.5 Electroanalysis Conditions

Electrochemical impedance spectroscopy (EIS) was performed prior to electrochemical measurements to enable the *iR*-drop compensation. All electrocatalytic measurements presented were fully *iR*-compensated. For Pt samples, LSV scans were conducted from 0.1 to −0.15 V versus RHE at a scan rate of 5 mV s^–1^. For Au samples, LSV scans were performed from 0.1 to −0.6 V versus RHE at a scan rate of 20 mV s^–1^. Multiple LSV data was recorded, and the 20th LSV is shown in the main text. The electrochemical stability of the samples was evaluated by using either extended LSV scans or chronopotentiometry (see Supporting Information). The experimental configurations and conditions for the LSV measurements are the same as those described above, unless otherwise specified in the caption of each corresponding figure. For chronopotentiometry, a current density of −10 mA cm^–2^ was applied based on the geometric surface area of 0.5 cm^2^ (0.7979 cm in diameter using C-A-BM_FC_HC-50-10x10, Redoxme, Sweden) against a Pt coil counter electrode (25 cm) with a Ag/AgCl reference electrode (3 M KCl, + 210 mV versus SHE; Redoxme, Sweden), and the *iR*-drop was fully corrected after the measurement.

### 2.6 Density Functional Theory Simulations

Fixed-spin^20^ van der Waals (vdW) corrected DFT simulations were executed via the Projected Augmented Wave (PAW) method as implemented in the VASP program.^21^ We used the PBE exchange–correlation (XC) functional,^22^ a 500 eV plane-wave energy cutoff, (0.1 eV, second order) Methfessel-Paxton electronic smearing,^23^ and a Γ-centered 14 *k*-point grid, numerically checked to yield energies converged to within 1 meV with respect to increased *k*-point samplings (20 *k*-points). To improve the description of the physiosorbed (in the absence of implicit or explicit solvent) final HER Tafel state, vdW corrections were applied based on Grimme’s parametrization.^24^

Following results in the recent HER literature,^25^ we primarily focused on the Tafel evolution from adsorbed H atoms on the FCC hollow and top positions on Au(111) and Pt(111), respectively. Full details of the computational methodology with additional results and discussion thereof can be found in the Supporting Information (see the section titled Supplementary DFT notes and results).

## 3 Results and Discussion

### 3.1 HER Electrocatalysis at PIM Au and Pt Electrodes

Pt/CoB/Ir superlattices are grown onto a thermally oxidized Si substrate deposited with a Ta seed layer (Figure 1) and capped with a conventionally nonferromagnetic electrocatalyst (either Au or Pt). Control samples where the Au or Pt is directly deposited on the Si substrate and Ta seed layer without the magnetic superlattices were also prepared. A full summary of the samples prepared is given in Table S1. Briefly, superlattice samples are prepared to give either PMA or IP magnetization by controlling the thickness of the CoB layers to examine the effect of stray magnetic fields during electrocatalysis with X-ray reflectivity being used to confirm layer structure and Magneto-Optic Kerr measurements confirming the desired magnetic properties (Figure 1). PMA samples are also prepared with a low density (LD) of large magnetic domains or with a high density (HD) of smaller magnetic domains to determine the effect of domain wall structure and stray field gradients on activity.^7^ The induced moment in the NM capping layer and hence the degree of spin polarization at the electrode/electrolyte interface will be dependent on the composition of the superlattice/NM interface, the spin diffusion length (λ_sd_), and the thickness of the NM capping layer (λ_c_)—see Table 1.^15–18^ Therefore, we have also prepared samples with Au and Pt capping layers of different λ_c_ to modulate the degree of spin polarization of the cap layer (Pt electrodes with λ_c_ = 5 and 10 nm, and Au electrodes with λ_c_ = 5, 10, 20 nm). For Au λ_sd_ is >30 nm at 300 K,^26,27^ while for Pt λ_sd_ is typically reported as <3 nm at 300 K, although we note values up to 11 nm have been reported in some cases.^17,18,27–30^ Atomic force microscopy and X-ray photoelectron spectroscopy analysis of the capping layer indicates that the samples exhibit atomistically flat surfaces, with roughness corresponding to approximately one or two atomic layers, and that following electrochemical HER studies, there is no roughening of the capping layer or migration of the magnetic sublayers (Figures S1 and S2; Pt RMS roughness: 0.13–0.19 nm).

In addition to being of significant scientific and practical interest, HER proceeds by well-understood, computationally tractable (vide infra), reaction mechanisms making it an ideal system to explore spin polarization effects here.^25^ We studied the Au and Pt electrodes in a common electrolyte, 0.5 M KHCO_3_. Recent studies have shown that HCO_3_^–^ acts as a proton donor during HER on Pt and Au and at moderate overpotentials such as those used here, HER is not primarily from H_2_O.^31,32^ This gives rise to onset potentials for HER in HCO_3_^–^, between those measured for hydronium and water reduction in Pt and Au. The use of a buffer as a proton donor also minimizes local pH gradients during HER and simplifies mechanistic analyses by removing the dominating effect of water dissociation during HER, which is required at pH values away from strongly acidic conditions. A full rationale for the choice of a buffered, near neutral (pH 8.8) electrolyte is included in the Supporting Information (Notes S1 and S2, Figures S3 and S4).

Linear sweep voltammetry (LSV) experiments on Pt in 0.5 M KHCO_3_ solution (Figures 2, S5) show an increase in HER current when the Pt (λ_c_ = 10 nm) is deposited on a PMA structure (i.e., Si/SiO_2_/Ta­(3.25 nm)/[Pt­(1.1 nm)/CoB­(1 nm)/Ir­(0.7 nm)]×3/Pt­(10 nm)), labeled here after Pt(10)-PMA-LD when compared to a control sample with a Pt cap deposited directly onto the nonmagnetic support (Pt(10)-NM). The HER activity is enhanced in the presence of magnetic superlattice. Specifically, at the *E*_min_ (i.e., −0.15 V versus RHE), the current density is −5.15 and −3.82 mA cm^–2^ for Pt(10)-PMA-LD and Pt(10)-NM, respectively. To demonstrate that the effect originating from the magnetic structure persists over time, we conducted an electrochemical stability test (Figures S6 and S7). A stable drop in the overpotential of 50 mV is achieved for HER at −10 mA cm^–2^ on Pt(10)-PMA-LD during extended electrolysis for hours. We propose that the change in HER activity is due to the presence of the magnetic substructure, either due to the generation of a spin-polarized Pt cap layer or as a result of the magnetic field generated by the underlayer.

**Figure 2:**
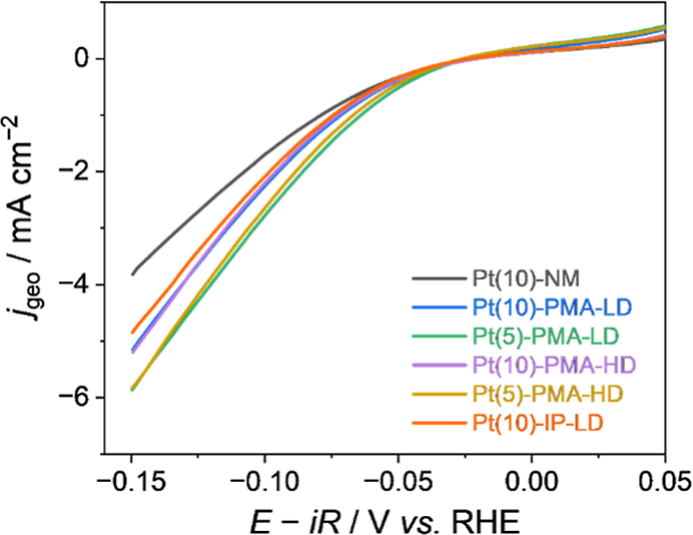
Cathodic HER tests on Pt-capped samples in 0.5 M KHCO_3_ solution (pH 8.8). Electrodes show a change in HER activity when the magnetic substructures are present. Pt(10)-NM refers to a 10 nm Pt layer deposited on a nonmagnetic Si/SiO_2_/Ta substrate (gray line). Blue, green, purple, and golden lines indicate PMA structures with differing cap thicknesses (λ_c_ in nm) and either a low (LD) or high (HD) density of magnetic domains (i.e., Pt­(λ_c_)-PMA-LD/HD). To assess the impact of stray magnetic fields, the activity of a Pt-capped sample with IP (in-plane) magnetic fields (orange, Pt(10)-IP-LD) is compared to an equivalent sample with an out-of-plane magnetic field (blue, Pt(10)-PMA-LD).

Proximity-induced magnetic moments have been measured for Pt with an exponential decrease in the X-ray magnetic circular dichroism signal with a decay-length scale of 1.8 ± 0.2 nm inside the Pt film.^18^ This decay of the proximity effect is in line with the reported Pt λ_sd_ values which are typically found to be <3 nm and a study examining the oxygen reduction reaction on Ag-capped Nd permanent magnets that showed that the impact of the magnet on the catalytic activity decreased with cap layer thicknesses that were significantly greater than λ_sd_.^17,18,27–30,33^ With the Pt(10)-PMA-LD electrode, although present, the degree of spin order will be limited due to λ_c_ > λ_sd_ (Table 1). Therefore, it can be anticipated that as λ_c_ is decreased, there will be a further increase in HER current if spin-polarization at the Pt surface either due to PIM or the presence of a spin-polarized current is the primary cause of the changes in Figure 2. Indeed, we measure a large increase in HER current density and a further positive shift in the onset potential for Pt(5)-PMA-LD samples when compared with both Pt(10)-PMA-LD and the Pt(10)-NM control sample. Specifically, the onset potential (defined as a potential at which the current density reaches −1 mA cm^–2^) is −64.1, –71.6, and −79.0 mV for Pt(5)-PMA-LD, Pt(10)-PMA-LD, and Pt(10)-NM, respectively.

In addition to being an excellent HER catalyst, Pt is also an effective hydrogen oxidation reaction (HOR) catalyst. During the repeated LSVs, some H_2_ will accumulate in the cell leading to a HOR current at positive potentials. Careful inspection of the LSVs in Figure 2 (see Figure S8 in Supporting Information for expansion) shows that the HOR activity increases in the order Pt(5)-PMA-LD > Pt(10)-PMA-LD > Pt(10)-NM indicating that HOR is also dependent upon the degree of spin-polarization.

Figure 2 shows that the HER current for Pt-capped samples does not depend on the size and density of magnetic domains with the activity of Pt(10)-PMA-LD ≈ Pt(10)-PMA-HD. Past studies on electrocatalytic OER at ferromagnetic electrodes have suggested that domain size/structure can impact catalytic activity as a result of domain wall scattering of spin polarized current,^6,7,14,34,35^ but the results here indicate that domain-wall scattering is not a significant factor controlling the activity of our samples. The magnetic field gradient present at the electrode surface is also strongly dependent on the domain size and density. The insensitivity of the HER current to domain structure also suggests that the impact of magnetohydrodynamic effects (Lorentz forces, ionic segregation)^10,12,36^ on the HER current is not significant. Confirming that stray magnetic fields are not the cause of the changes in the HER current is the observation that samples where the magnetic field is aligned parallel (in-plane) to the electrode surface (Pt(10)-IP-LD) have the same HER activity as those prepared with the magnetic field perpendicular to the electrode surface (Pt(10)-PMA-LD, Figure 2). The in-plane samples have minimal stray field present at the Pt/electrolyte interface, but they will have the same degree of spin polarization as the PMA sample (see Table 1). Therefore, the equivalent activity of Pt(10)-IP-LD and Pt(10)-PMA-LD for HER confirms that spin-polarization of the Pt is the cause of the increase in HER current.

To further test the hypothesis that PIM can be used to control electrocatalytic activity, we examined HER on Au (Figure 3). On Au-capped electrodes, the presence of the PMA structure leads to a pronounced, λ_c_-dependent, decrease in HER activity. The HER activity follows the trend Au(10)-NM > Au(20)-PMA-LD > Au(10)-PMA-LD > Au(5)-PMA-LD in the KHCO_3_ electrolyte. Specifically, at the *E*_min_ (i.e., −0.6 V versus RHE), the corresponding current density values are −1.08, –0.72, −0.54, and −0.31 mA cm^–2^, respectively. To demonstrate that the observed effect persists under cathodic conditions and when a different magnetic substrate is used, we have conducted extended cycling tests (Figures S9 and S10) and an additional set of LSV measurements using Co/Pt superlattice samples instead of a Pt/CoB/Ir system (Figure S11). The results show that the effect arising from the PMA structure is sustained throughout the extended loops and that the Co/Pt system exhibits the same λ_c_-dependent HER activity trend as seen in the Pt/CoB/Ir system. The marked difference in direction of change in activity on Au spin-polarized electrodes (decrease in HER activity, Figure 3) compared to Pt electrodes (increase in HER activity, Figure 2) is rationalized through DFT studies below. To quantify the impact of the spin-polarization on HER current, we have measured the applied potential required to reach −0.1 mA cm^–2^ ( $E_{-0.1mAcm^{-2}}$ ) on Au and find that it is ∼39 mV more negative for Au(10)-PMA-LD compared to Au(10)-ND. In contrast, on Pt, we measure a smaller ∼4 mV, positive, shift in potential to reach −0.5 mA cm^–2^ comparing Pt(10)-PMA-LD to Pt(10)-ND. A higher current density was chosen as the benchmark for Pt to remove the potential contributions from changes in the HOR activity induced by the magnetic underlayers. Figure 4 plots the dependence of $E_{-0.1mAcm^{-2}}$ (Au) and $E_{-0.5mAcm^{-2}}$ (Pt) on λ_c_ and a reasonable fit to a single exponential decay is seen for both metals—the statistical persuasiveness of our approach is reinforced using log­(*j*) versus λ_c_ in Figure S12. There is significant uncertainty in the fits, but notably, the rate of drop-off of the effect of λ_c_ on electrocatalytic activity for Pt (5 ± 2 nm) is greater than that for Au (14 ± 8 nm), in-line with the trend in reported spin-diffusion lengths (λ_sd_: Pt typically <3 nm, Au >30 nm both at 300 K).^17,18,27–30^ This is again supporting the conclusion that spin-polarization of the capping metal is leading to the measured change in HER rates.

**Figure 3:**
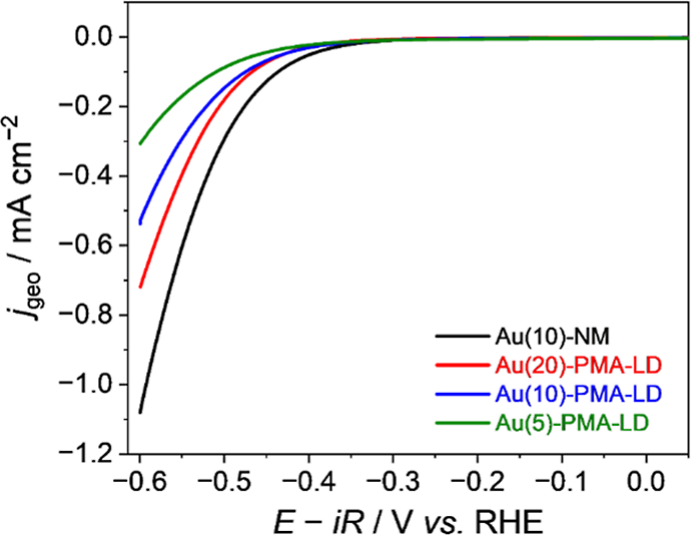
Cathodic HER tests on Au-capped samples in 0.5 M KHCO_3_ solution (pH 8.8). Electrodes show a change in HER activity when the magnetic substructures are present. Au(10)-NM refers to a 10 nm Au layer deposited on a nonmagnetic Si/SiO_2_/Ta substrate (black line). Red, blue, and green lines indicate PMA structures with differing cap thicknesses (λ_c_ in nm) and a low (LD) density of magnetic domains (i.e., Au­(λ_c_)-PMA-LD).

**Figure 4:**
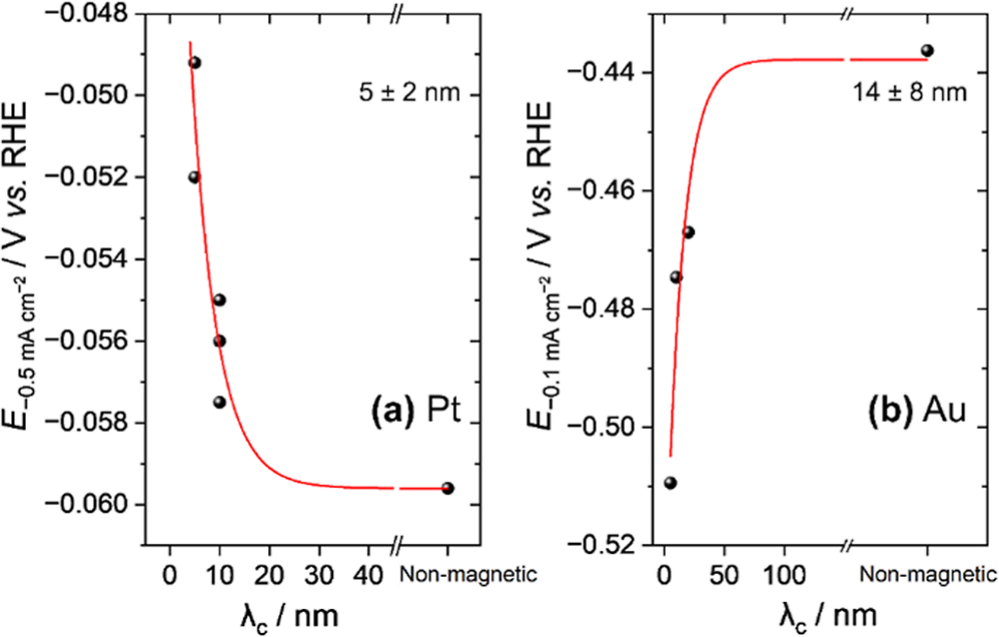
Potential required to reach −0.5 mA cm^–2^ current density on Pt-capped electrodes (a) and −0.1 mA cm^–2^ on Au-capped electrodes in 0.5 M KHCO_3_ during LSV measurements (b). The red lines show a fit to a single exponential decay function with the decay distance quoted.

### 3.2 Mechanism of PIM-Induced Control of HER

To understand the contrasting behavior of the Au and Pt surfaces during HER, fixed-spin Density Functional Theory (DFT)^20^ simulations on two idealized models, namely, Au(111) and Pt(111) surfaces, were carried out. Following recent literature on the HER,^25^ we focused on the evolution of H_2_ from two H atoms (2H) adsorbed on the FCC-hollow and top positions of Au(111) and Pt(111), respectively. In line with this, the experimentally observed preferred orientation of the Au and Pt films used in the electrochemical experiments above is (111), see Figure S13. Free-energy barriers for the Tafel step (i.e., a chemical step where two adsorbed H atoms react and desorb as a H_2_) were computed as a function of the spin-polarization (measured as Bohr magneton, μ_B_, per metal-atom) of the catalytic surface in the range 0–0.4 μ_B_ beyond which instabilities due to the use of a fixed slab in-plane periodicity started to manifest, especially for transition-state searches. Recent experimental studies^18,37–39^ of proximity magnetism on analogous Pt-capped PMA substrates (Co_25_Fe_75_) have reported magnetic moments in the range of 0.2–0.7 μ_B_/Pt-atom, although these decay sharply, in line with a short λ_sd_ value, a persistent magnetism in Pt atoms is measured at 3 nm,^18^ and the residual spin-polarization at the electrolyte-exposed Pt surface in the samples used here should be nonzero. For Au in equivalent structures, the reported magnetic moments are roughly half that measured in Pt (making values of 0.1–0.35 μ_B_/Au-atom) and the depth-damping will be significantly decreased,^18,37–40^ making our chosen spin-polarization range a reasonable approximation of the experimental system.

Typically studies on electron spin-polarized surfaces for electrocatalysis have focused on the possible generation and accumulation of spin-polarized surface reaction intermediates,^19^ but it is important to recognize that the adsorption (free) energy of a surface species on a metal can be strongly affected by spin-polarization of the surface, even without the surface species being itself spin-polarized, providing a route to modulate catalytic activity.^2^ Here, we find that there is no evidence of transfer of magnetism to the absorbed H atoms (2H) or to the (H–H)* transition state at either the Au or Pt surface (Table S5 in Supporting Information, Figure S14 onward and related discussion). Despite this, the calculated change of the HER Tafel free-energy barrier on Au(111) is found to be significant, revealing a strong dependence on the surface spin-polarization which can be assigned to substrate-mediated changes in adsorption free energies of the metalized surface species (Figure 5). As the spin-polarization (atomic magnetic moment) of the Au atoms is increased, the barrier for the Tafel step increases by up to ∼140 meV, in-line with the large decrease in HER activity for the samples with the magnetic structures (Figure 5e). For Pt(111), the effect of different spin-polarizations is smaller, but notably the free-energy barrier on Pt(111) decreases upon spin-polarization, ∼30 meV (Figure 5f). This result is also in-line with the experimental HER activity where an increase in activity for the Pt samples with magnetic layers was measured, but the relative change compared to the control nonmagnetic sample was smaller than that seen with the Au sample set.

**Figure 5:**
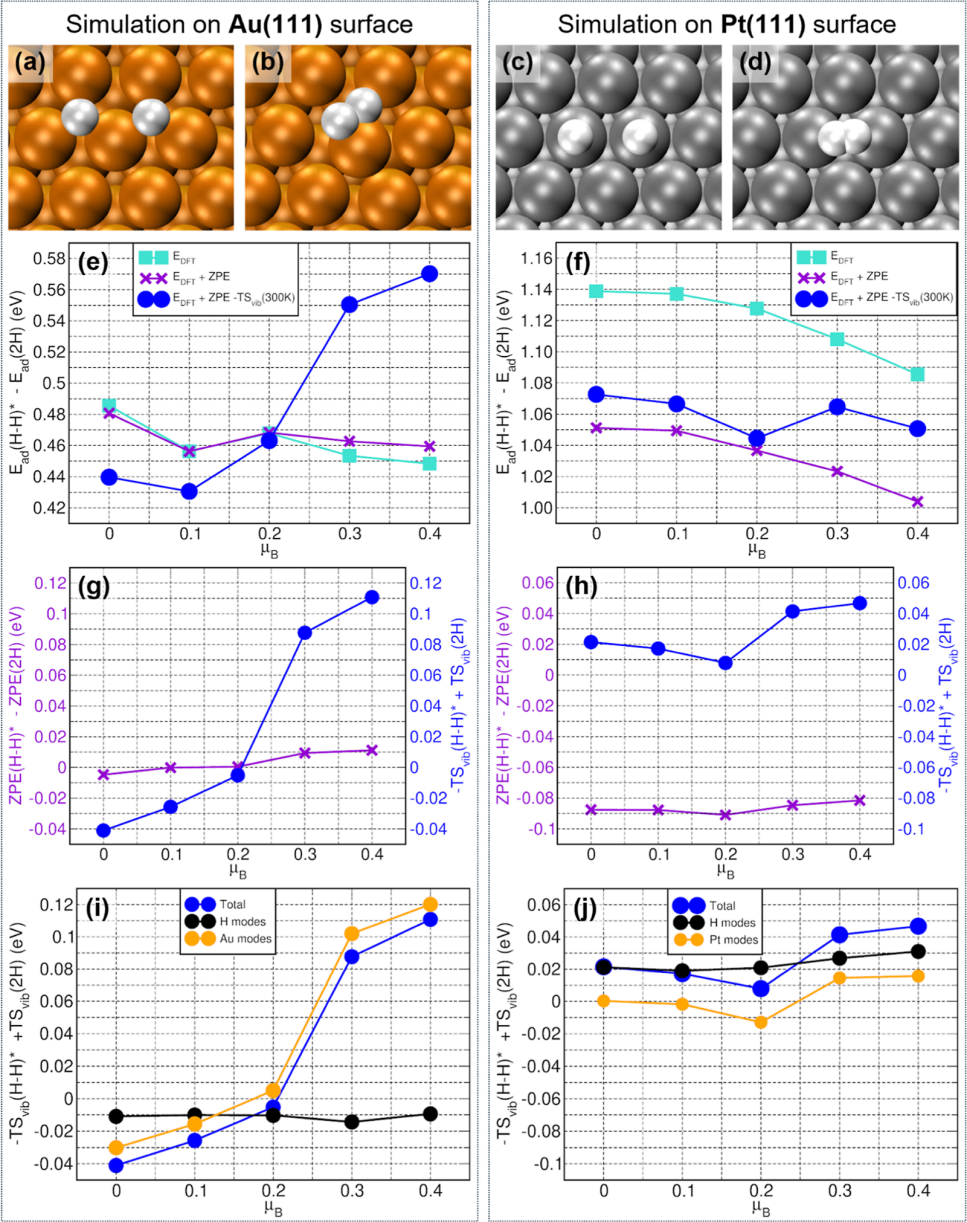
Optimized initial (2H) and transition state (H–H)* geometries for the Tafel reaction step on Au(111) (a,b) and Pt(111) (c,d). Au: orange, Pt: gray, H: white. Calculated reaction barriers based on DFT energies only (*E*_DFT_), *E*_DFT_ + ZPE, and free energies (E_DFT_ + ZPE – *TS*_vib_, *T* = 300 K) on (e) Au(111) and (f) Pt (111). ZPE and −*TS*_vib_ contributions to reaction free-energy barriers on (g) Au(111) and (h) Pt (111). H atom and metal-slab resolved contributions to −*TS*_vib_ barriers on (i) Au(111) and (j) Pt(111).

Given the opposite trends measured on Au and Pt for the HER Tafel step involving the same 2H and (H–H)* species, it is clear a theoretical model based on systematic upward energy shift and ensuing depopulation of the metal–H antibonding orbital for spin-polarized substrates (as proposed in ref 2 for intrinsically ferromagnetic surfaces) cannot explain the present experiments. An extension of the model is accordingly needed.

As seen in Figure 5f, barriers calculated based on DFT energies alone *(E*_DFT_ trace), neglecting vibrational zero-point energy (ZPE) and entropic contributions (−*TS*_vib_; *T*: temperature; *S*_vib_: vibrational entropy), do suggest a decrease of the Tafel barrier with spin-polarization on Pt(111) in line with experiments. However, the same level of theory applied to Au(111) does fail to reproduce even qualitatively the experimentally observed increase of the Tafel barrier with spin-polarization. This is not totally unexpected as free-energy differences accounting for vibrational ZPE and entropic contribution, not DFT-ones based on electronic structure only, determine reaction barriers.^41,42^ Notably, vibrational ZPE and entropies on magnetically proximitized metals could be in principle as or more important than those for the nonmagnetic counterpart extensively considered in the HER on Au or Pt literature.^25^ Indeed, further analysis (Figure 5g,h) enables assignment of the dominating factor for the computed changes in the Tafel barrier to the vibrational entropy for the magnetically least susceptible metal (Au, with a molar magnetic susceptibility χ_m_ of −28 × 10^–6^ cm^3^ mol^–1^)^43^ and ZPE for the magnetically most susceptible one (Pt, χ_m_ = +193 × 10^–6^ cm^3^ mol^–1^).^43^ Not unexpectedly given the profoundly lower vibrational energy with respect to H-containing modes, the changes in the entropic contribution (−*TS*_vib_) to the barriers as a function of the Au(111) spin-polarization (Figure 5g) are dominated by the Au modes (phonons). However, the opposite holds true for the Pt(111) results. The changes in the Tafel barrier due to ZPE changes are approximately −0.09 eV, a significantly larger change than the one due to the entropic (−*TS*_vib_) contribution (approximately 0.01–0.045 eV) (Figure 5h). In addition, as seen in Figure 5i, the FCC hollow H adsorption site on Au(111) results in larger spin-polarization-induced phonon-softening (thence increase of *S*_vib_ and decrease of −*TS*_vib_) with respect to the HER Tafel transition state, leading to an overall increase of the resulting barrier. By comparison to the results for Au(111), the spin-polarization effects on the differences in −*TS*_vib_ between the HER Tafel initial and transition states on the Pt(111) are effectively negligible (Figure 5j).

Here, it is important to note that, as seen in Figure S19, whereas the d-band center on Au(111) does monotonically increase with spin-polarization (in line with results from ref 2), this does not hold for Pt(111). Furthermore, whereas the difference in d-band center between (H–H)* and 2H on Au(111) is overall increasing with spin-polarization, the trend does not reflect what was calculated in terms of (DFT) adsorption energies (*E*_ad_, Figure S18) and barriers (Figure 5e). For the Tafel HER barrier to increase on Au(111), vibrational entropy (also of the Au layers) must be considered in addition to DFT energies (due to the electronic structure in a fixed nuclear configuration). For Pt(111) too, it is not possible to correlate the calculated changes in the d-band center with slab spin-polarization to the experimentally observed modification in Tafel HER currents, thence barriers. Plot of *E*_ad_ as a function of the calculated d-band center for each considered system (Figure S20 in the Supporting Information) reiterates the conclusion on the change in the Tafel HER barrier not being dominated by electronic structure or molecular orbital bonding factors alone. This conclusion is further strengthened by analysis of H-projected electronic density of states (PDOS), provided in Figure S21: whereas for Au(111), the occurrence of spin-polarization reveals the, expected,^2^ upward energy shifts of the (P)­DOS, the changes on Pt(111) are substantially reduced. Thus, arguments on increased bonding to the metal in the presence of spin-polarization due to reduced population of antibonding metal–adsorbate orbitals^2^ appear not to be rigidly applicable to the present HER on Au(111)/Pt(111) cases.

Combined with the supplementary analysis and discussion in Figures S23–S25, altogether these results point to a complex dependence of the HER Tafel barrier on the interplay between the system- and state-dependent responses to surface spin-polarization of (i) adsorption geometries and energies, (ii) ZPEs, and (iii) metal-surface dominated vibrational entropies, thence free energies, with (i)–(iii) inevitably linked to the occurrence of, potentially intermediate-dependent (e.g., Supplementary Figure 15 of ref 44), adsorbate metallization. While not addressed by the present results on predominantly (111) substrates (Figure S13), we speculate metal–surface faceting, i.e., terminations different from the FCC(111) surfaces studied here, may also play a role in additionally tuning (HER) activity for a given electrocatalyst composition. We hope the present results will stimulate further cross-disciplinary research in this extremely vast, and ultimately multiscale, multiphysics catalyst optimization space.

## 4 Conclusions

Here, we have demonstrated how routine structures from the spintronics community can be used in catalysis studies to selectively (de)­stabilize species involved in the Tafel HER step through PIM of conventionally nonmagnetic materials. A key advantage of our approach is also that by control of the thickness of the layers of the magnetic materials, we are able to carry out experiments where there will be negligible stray magnetic field at the electrode/electrolyte interface. This allows us to confirm that magneto-hydrodynamic effects, a likely cause of the changes in catalytic activity at many studies where a magnetized electrode is used,^10^ are minimal, leading to the conclusion that activity changes here are due to spin-polarization of the electrode surface. Initially, we focus on a single model electrocatalytic reaction (HER) at Au and Pt surfaces, but the method is flexible to both the capping metal (being applicable across a vast range of nonferromagnetic materials) and the reaction studied. Therefore, we anticipate that this chemo-spintronics approach, where spintronic structures are used to modify the chemical catalytic activity of existing materials, has the potential to circumvent scaling relationships across photo-, thermal-, and electrocatalysis.

## Supplementary Material



## Notes

Characterization and electrochemical study of the samples in addition to full details on the DFT calculations and full list of abbreviations, acronyms, and symbols (PDF)

## ⊥

Department of Chemical Sciences, University of Padua, Padova 35131, Italy
