# Supplementary material for: Spin-Polarized Nonferromagnetic Surfaces for Electrocatalysis: Chemo-Spintronics
Source: J Am Chem Soc. 2025 Dec 19;148(1):967–75. doi: 10.1021/jacs.5c16824 (PMC12814321; doi:10.1021/jacs.5c16824)
Supplement: Supplementary file 1 [file ja5c16824_si_001.pdf]

Supporting Information

Spin-Polarized Non-Ferromagnetic Surfaces for Electrocatalysis: Chemo-Spintronics

Hansaem Jang,[1] Daniel Roe,[2] Harry E. Taylor,[3] Emiliano Poli,[4] Alex S. Walton,[3] Gilberto Teobaldi,[4*] Oscar Cespedes,[2*] Alexander J. Cowan[1*]

[1]Department of Chemistry and Stephenson Institute for Renewable Energy, University of Liverpool, Liverpool L69 7ZF, UK

[2]School of Physics and Astronomy, University of Leeds, Leeds LS2 9JT, UK

[3]Department of Chemistry and Photon Science Institute, The University of Manchester, Manchester M13 9PL, UK

[4]Scientific Computing Department, Science & Technology Facilities Council UKRI, Rutherford Appleton Laboratory, Didcot OX11 0QX, UK

*Corresponding authors: gilberto.teobaldi@stfc.ac.uk; o.cespedes@leeds.ac.uk; acowan@liverpool.ac.uk

List of abbreviations, acronyms and symbols

(*arranged in alphabetical order, with Greek letters listed first, followed by Latin letters*)

ε_d	d-band centre
η	overpotential
θ	angle (in X-ray diffraction)
θ	coverage (in DFT calculations)
λ_c	cap layer thickness
λ_{CoB}	cobalt boride layer thickness
λ_{sd}	spin diffusion length
μ_B	Bohr magneton
ν_i	vibrational frequency
a.u.	arbitrary unit
**B**	magnetic flux density
CoB	cobalt boride
DFT	density functional theory
E	energy (in DFT calculations)
E	potential (in electrochemistry)
$E_{2H/(H\text{-}H)*}$	(free) energy of the slab with the Tafel initial/transition state
E_{ad}	adsorption energy
E_{DFT}	DFT energy
E_F	Fermi level
E_{H2}	energy of one H_2 molecule optimised in vacuo
E_{min}	lower potential limit
E_{slab}	(free) energy of the bare optimised slab
EIS	electrochemical impedance spectroscopy
FCC	face-centred cubic
**H**	magnetic field
h	Planck constant
HCP	hexagonal close-packed
HD	high density of maze domains

HER	hydrogen evolution reaction
I	intensity
i	current
IP	in-plane
j	current density
j_{geo}	geometric current density
k_B	Boltzmann constant
LD	low density of maze domains
LSV	linear sweep voltammetry
ML	molecular layer
$n_d(E)$	d-states resolved density of states
NM	non-magnetic
**P**	spin polarisation
PAW	projected augmented wave
PBE	Perdew–Burke–Ernzerhof
ph-DOS	phonon density of states
PIM	proximity-induced magnetism
PMA	perpendicular magnetic anisotropy
R	resistance
RHE	reversible hydrogen electrode
RMS	root-mean-square
S_{vib}	vibrational entropy
SOC	spin–orbit coupling
T	temperature (in DFT calculations)
TrS	transition state
vdW	van der Waals
XC	exchange-correlation
XPS	X-ray photoelectron spectroscopy
z	distance
ZPE	zero-point energy

Supplementary experimental notes

Note S1. To enable comparison between the Au and Pt capped electrodes it is highly desirable to operate in a common electrolyte. HER via hydronium reduction, particularly on Pt, rapidly gives rises to pH gradient generation and the very high current densities at even moderate overpotentials lead to complications from bubble formation/removal. Possible hydrodynamic contributions (e.g. impact on bubble behaviour, pH gradient) are of particular concern here due to the demonstration that many studies on magnetic electrodes may be reporting results where the current response is dominated by magnetohydrodynamics.[1, 2] Rotating disk electrodes can minimise mass transport effects, but the complex nature of our electrode prevents this experiment. Therefore, we have avoided carrying out experiments in acidic solutions.

In alkaline electrolyte on Pt and Au water dissociation becomes limiting. Water dissociation is extremely sensitive to electrolyte composition (e.g. presence and nature of ionic species and concentration at the surface)[3, 4] which again may complicate the planned studies on the surface electrocatalytic mechanism occurring here. Therefore, we have used HCO_3^- as a simple proton donor to enable HER. This provides a route to study the impact of spin polarisation on the HER mechanism, in particular the H–H formation process whilst minimising the impact of other external effects.

The mechanisms of HER production from HCO_3^- have been studied in detail.[5, 6] At the concentrations used here (0.5 M) HER is shown to be insensitive to the nature of the cation at the potentials used here on both Pt an Au, indicating that H* formation is not via the water dissociation during the Volmer step. It is also demonstrated that the use of high concentration (0.5 M) solutions is effective in minimising pH gradients in the current/potential windows used here. In-line with past studies we find that the use of HCO_3^- leads to an HER onset at ca. −0.05 and −0.4 V (Pt, Au), between that of the same electrodes when they are operating in acidic and alkaline water.[5, 6]

Note S2. Tafel analysis is commonly used to explore HER rate determining steps within the community. However it is important to note that the suitability of classical Butler-Volmer kinetics to model HER kinetics, and hence the Tafel analysis has been disputed recently[7] with growing evidence that the potential dependence of the transition state exists.[8] Furthermore the Tafel approximation typically excludes the low overpotential region and for more active catalysts (e.g. Pt as used here) the linear region of the Tafel slope is very limited due to a combination of mass transport limitations and changes in charge transfer coefficient.[9] Tafel plots are presented for the Au and Pt capped samples, and indeed whilst these do show some changes in the Tafel slope indicative of changes in mechanism/barrier, on the basis of the discussion above we do not attempt to quantify these or discuss further (see Fig. S3–S4 below).

Supplementary experimental tables

Table S1: Sample structure for the electrodes used in the main study. All samples were grown on a Si/SiO_2(100 nm) substrate. An alloy target of $Co_{68}B_{32}$ is used to grow the CoB layers.

Sample Name	Sample Structure	Domain Type (Polar/OOP Kerr Microscopy)
Pt(10)-PMA-HD	Substrate/Ta(3.25 nm)/[Pt(1.1 nm)/CoB(1 nm)/Ir(0.7 nm)]×5/ Pt(9.9 nm)	Small Maze
Pt(5)-PMA-HD	Substrate/Ta(3.25 nm)/[Pt(1.1 nm)/CoB(1 nm)/Ir(0.7 nm)]×5/ Pt(5 nm)	Small Maze
Pt(10)-PMA-LD	Substrate/Ta(3.25 nm)/[Pt(1.1 nm)/CoB(1 nm)/Ir(0.7 nm)]×3/ Pt(9.9 nm)	Big Domains
Pt(5)-PMA-LD	Substrate/Ta(3.25 nm)/[Pt(1.1 nm)/CoB(1 nm)/Ir(0.7 nm)]×3/ Pt(5 nm)	Big Domains
Pt(10)-NM	Substrate/Ta(3.25 nm)/ Pt(9.9 nm)	Non-Magnetic
Pt(10)-IP-LD	Substrate/Ta(3.25 nm)/[Pt(1.1 nm)/CoB(1.8 nm)/Ir(0.7 nm)]×5/ Pt(9.9 nm)	IP Magnetisation
Au(20)-PMA-LD	Substrate/Ta(3.5 nm)/[Pt(1.1 nm)/CoB(0.7 nm)/Ir(0.8 nm)]×3/ Au(20 nm)	Big Domains
Au(10)-PMA-LD	Substrate/Ta(3.5 nm)/[Pt(1.1 nm)/CoB(0.7 nm)/Ir(0.8 nm)]×3/ Au(10 nm)	Big Domains
Au(5)-PMA-LD	Substrate/Ta(3.5 nm)/[Pt(1.1 nm)/CoB(0.7 nm)/Ir(0.8 nm)]×3/ Au(5 nm)	Big Domains
Au(10)-NM	Substrate/Ta(3.5 nm)/ Au(10 nm)	Non-Magnetic

Supplementary experimental figures

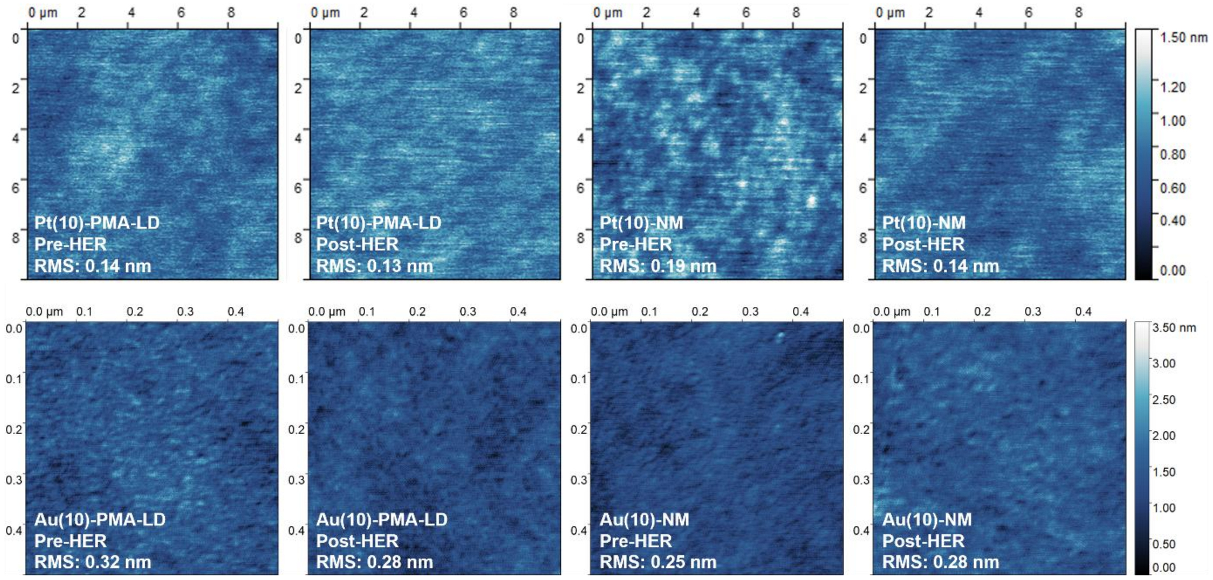

Figure S1. Atomic Force Microscopy maps of (*top*) Pt and (*bottom*) Au, recorded prior to and post-HER studies in 0.5 M $KHCO_3$. The root-mean-square (RMS) roughness of the samples is estimated to be below or approximately 0.3 nm, indicating an atomically flat surface with a roughness corresponding to one or two atomic layers. The comparison of RMS roughness between pre- and post-HER samples shows no evidence of roughening during HER. Based on this result, we deduce that the electrochemical surface area and geometric surface area are comparable. However, estimating the electrochemical surface area using atomic force microscopy may not be optimal as the surface morphology can dynamically change under electrochemical conditions. Therefore, we additionally conducted a series of voltammetry measurements to electroanalytically demonstrate that surface restructuring was negligible. As shown in Fig. S10, the voltammetry is consistent between the first and repeated runs, which indicates that any change in surface area during the experiments was minimal. On the basis of Fig. S1 and S10, we concluded that the electrochemical surface area and geometric surface area were maintained in the systems studied in our work. Please note that the "Pre-HER" and "Post-HER" samples are not observations of the same sample before and after the experiment, but rather a comparison between a post-mortem sample and a pristine sample with the same sample structure.

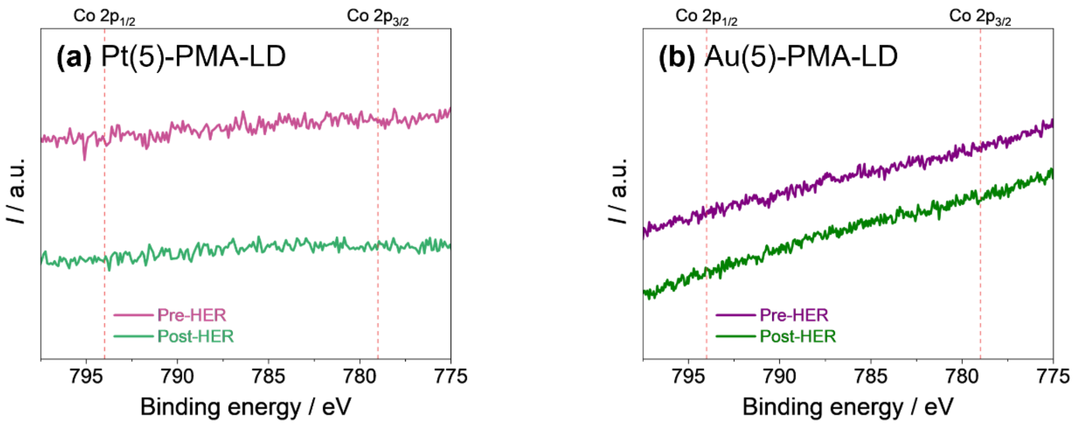

Figure S2. X-ray Photoelectron Spectroscopy (XPS) analysis of (a) Pt-capped and (b) Au-capped samples, pre- and post-HER studies in 0.5 M $KHCO_3$. The lack of detection of Co by XPS supports the conclusion that effective coverage of the magnetic structure is achieved and that there is no migration of the magnetic sub-layers to the catalyst surface during electrolysis. We focus on the sample capped with 5 nm Pt or Au layer as the thinnest capping layer would be anticipated to show the lowest stability/resistance to underlayer migration and roughening. Please note that the “Pre-HER” and “Post-HER” samples are not observations of the same sample before and after the experiment, but rather a comparison between a post-mortem sample and a pristine sample with the same sample structure.

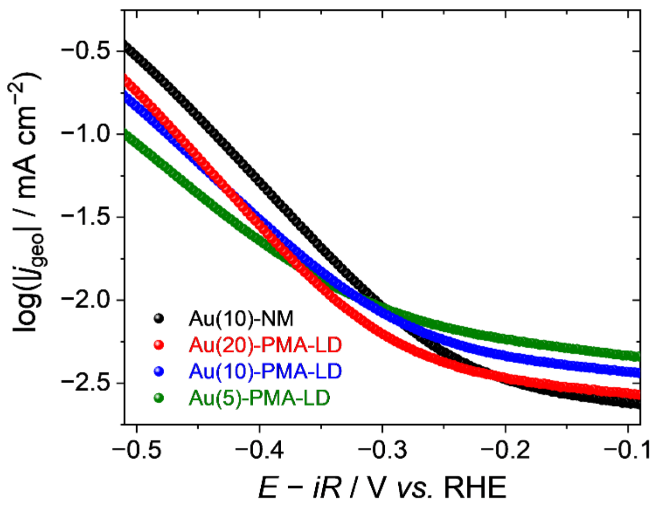

Figure S3. Tafel plot of the LSV data presented in the main test of the Au-capped samples in 0.5 M $KHCO_3$.

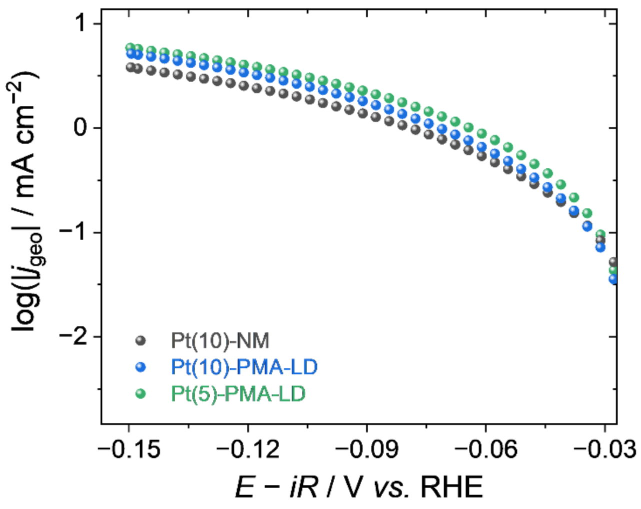

Figure S4. Tafel plot of the LSV data presented in the main test of the Pt-capped samples in 0.5 M $KHCO_3$.

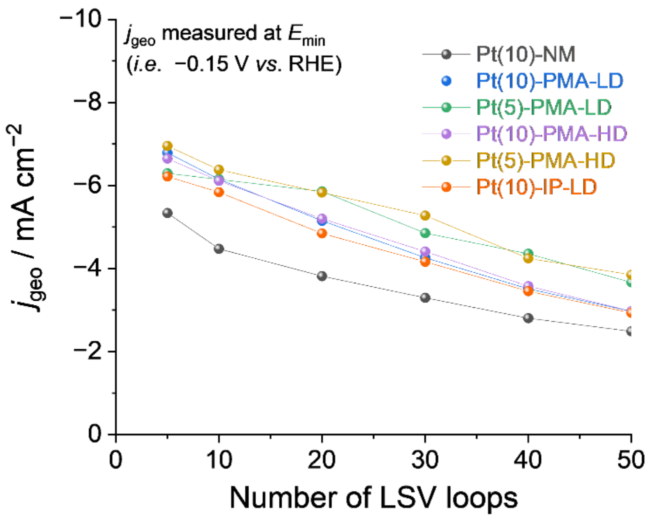

Figure S5. Measured current density following repeated LSVs for the Pt-capped samples in 0.5 M $KHCO_3$ at −0.15 V vs RHE. The data presented in the main paper is taken from LSV 20. The gradual decrease in current density indicates that the H_2 bubbles accumulate on the surface, blocking the active sites – see discussion in Fig. S7.

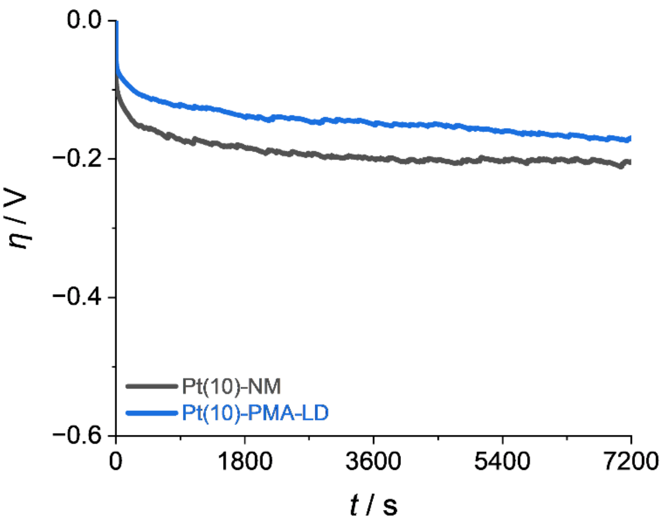

Figure S6. Chronopotentiometry for Pt samples held at −10 mA cm^{-2} in 0.5 $KHCO_3$ demonstrating the stable nature of the enhanced HER activity in the presence of the magnetic layers.

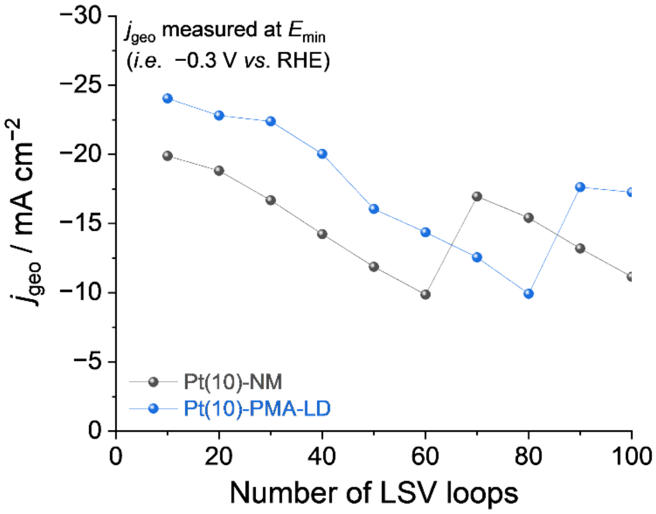

Figure S7. Measured current density following repeated LSVs for the Pt capped samples in 0.5 M $KHCO_3$ at −0.3 V vs RHE. Compared to Fig. S5, the lower potential limit (E_{min}) is extended by 0.15 V, and the number of LSV loops is doubled to enable vigorous occurrence of the HER, thereby facilitating the formation, growth, accumulation, and detachment of H_2 bubbles on the electrode surface. Referring to Fig. S5 (or its equivalent for Au, Fig. S9), one may wonder why the activity decreases over the course of repeated measurements. We attribute the gradual decrease in current density to the accumulation of H_2 bubbles on the surface, blocking the active sites. To test the assumption, we performed the experiment using modified electroanalytical parameters, as shown in Fig. S7 (E_{min} = −0.3 V vs RHE), where the HER can occur more vigorously compared to Fig. S5 (E_{min} = −0.15 V vs RHE). Similar to Fig. S5, a gradual decrease in current density is initially observed during the early LSV loops. During the extended LSV loops, H_2 continues to grow and accumulate on the surface, eventually detaching around 70–80 loops, which restores the current density. This result confirms that the decrease in current density arises from the accumulation of H_2, rather than from the degradation of the sample structures or conditions. A further systematic study, presented in Fig. S10, reinforces the conclusion drawn here.

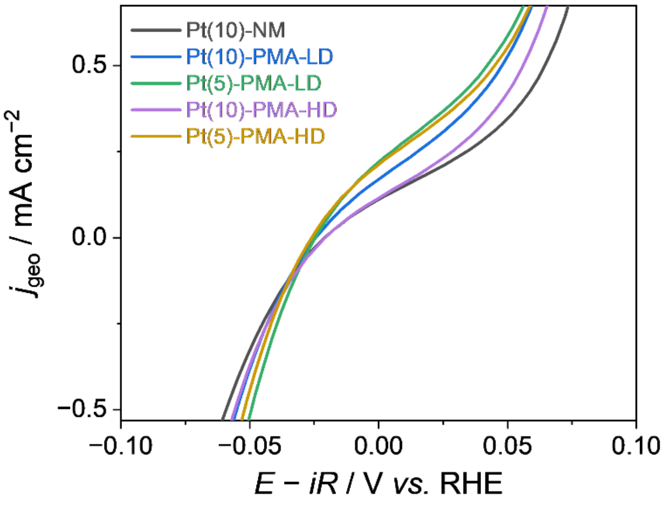

Figure S8. Expanded LSV for Pt-capped samples in 0.5 M $KHCO_3$ showing how the oxidation current assigned to HOR changes with electrode structure.

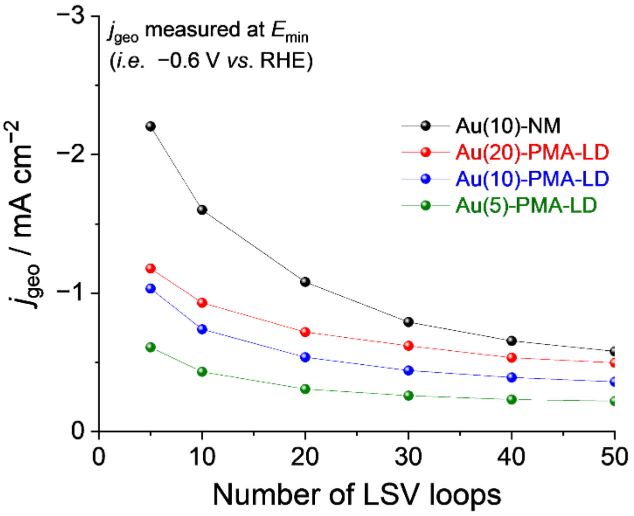

Figure S9. Measured current density following repeated LSVs for the Au capped samples in 0.5 M $KHCO_3$ at −0.6 V vs RHE. The data presented in the main paper is taken from LSV 20. The gradual decrease in current density indicates that the H_2 bubbles accumulate on the surface, blocking the active sites – see discussion in Fig. S7.

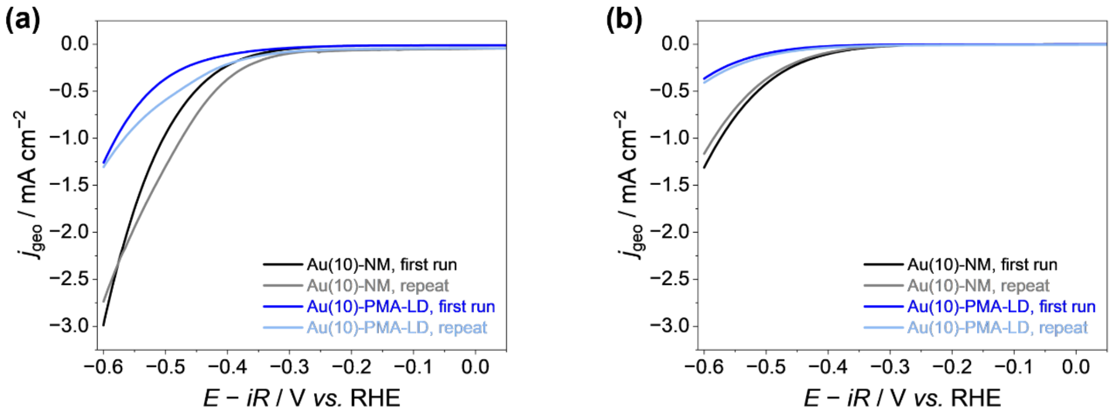

Figure S10. Cathodic HER tests on Au-capped samples in 0.5 M $KHCO_3$ solution (pH 8.8), measured at the (a) 1st and (b) 20th LSV cycles. Over 100 cycles of LSV were performed during the first run (black and blue lines), after which repeat experiments (grey and light blue lines) were carried out in fresh electrolyte, following the removal of H_2 bubbles from the electrode surface and rinsing of the electrodes. The electrodes demonstrate consistent performance between the first and repeated experimental runs, confirming the stable nature of the sample structures and conditions even after cathodic polarisation.

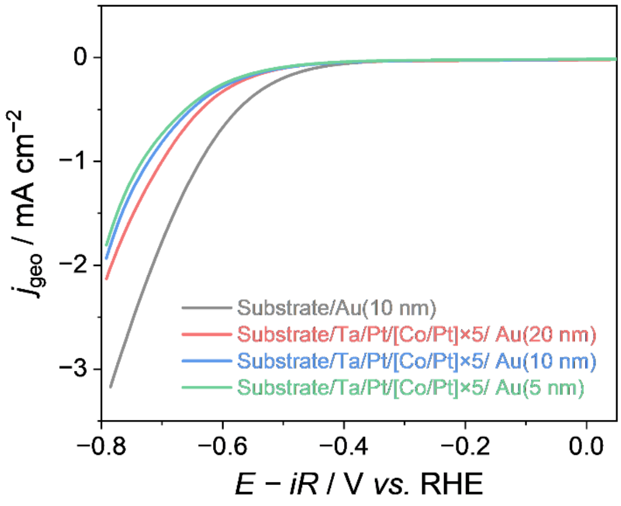

Figure S11. Cathodic HER tests on Au-capped samples in 0.5 M $KHCO_3$ solution (pH 8.8), measured at the 5th LSV cycle, in a H-type cell (R-A-ECSYNTH_E/S, Redoxme, Sweden) with a surface area of 0.09 cm^2. Here, the sample structures and experimental conditions differ from those in Fig. 3 (cf. Table S1), and the substrate is p-type Si rather than Si/SiO_2. Despite the differences, the same trend is observed in both Fig. 3 and Fig. S11.

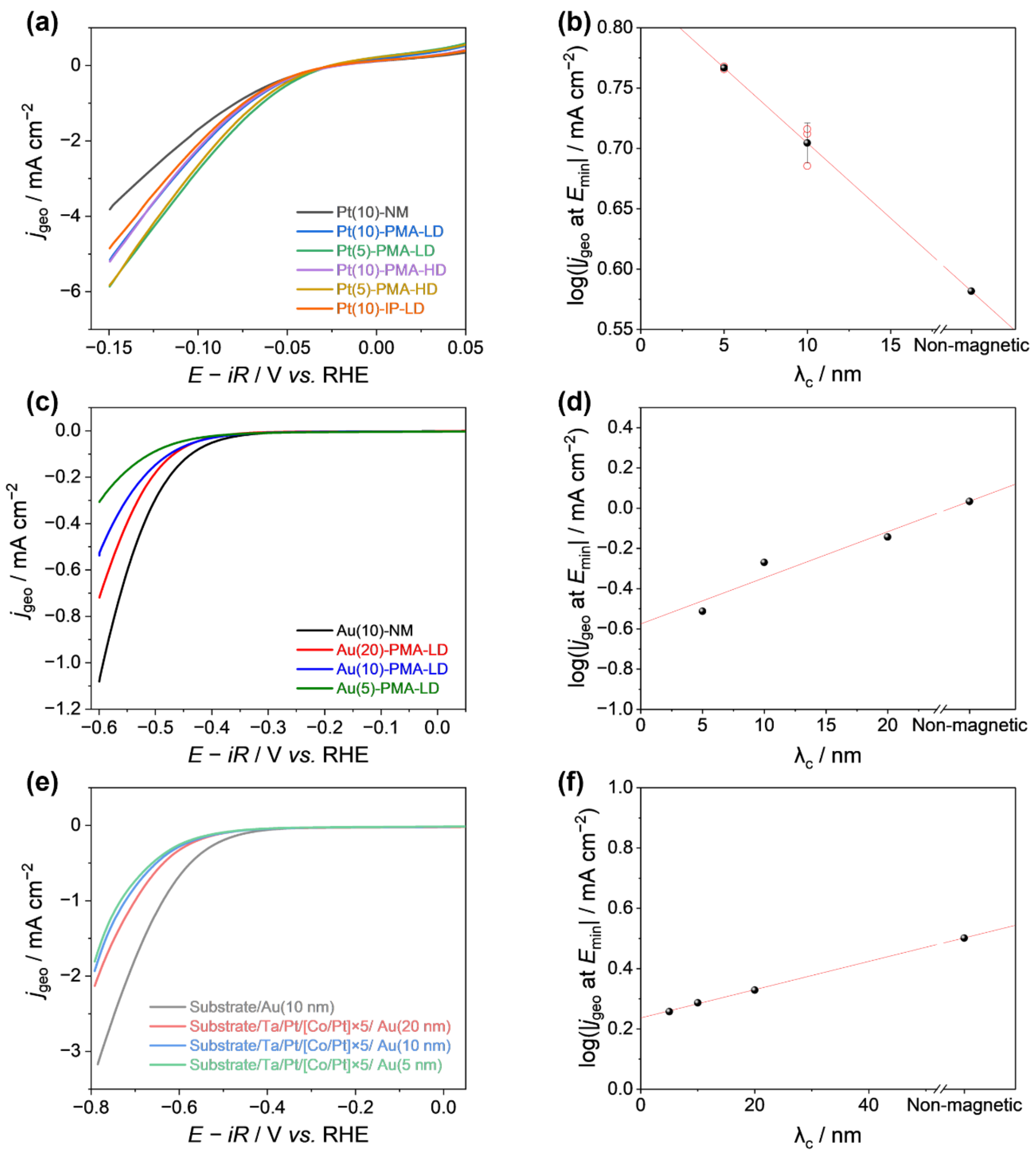

Figure S12. Regression analyses for the (a,b) Pt system in Fig. 2, (c,d) Au system in Fig. 3, and (e,f) Au system in Fig. S11. Given the statistically non-negligible error observed in the original Au system (panel d, $R^2 = 0.87$), another set of Au system was investigated to strengthen our conclusion and minimise potential uncertainty (panel f). In the new Au system, a linear correlation is observed between λ_c and log(j) with a $R^2 = 0.99$; therefore, an exponential relationship between λ_c and j can be inferred, in good agreement with the expected decay behaviour of spin polarisation through the Au cap layer, confirming the strong interdependence between the investigated parameters.

Supplementary DFT notes

Two H atoms were modelled in a (111)-4×2 orthorhombic cell whose in plane-periodicity, defined on the basis of DFT-optimised bulk structure, was and 11.540×9.994 Å^2 [Au(111)] and 11.247×9.587 Å^2 [Pt(111)]. The Au(111) and Pt(111) slabs had five layers, the bottommost of which was kept fixed during geometry optimisations. Following earlier computational results and benchmarks on DFT simulation of Tafel HER on Pt slabs,[10] we did not include explicit solvent molecules in the simulations. Geometry optimisations were converged to within atomic force tolerance of 0.02 eV/Å. Transition state (TrS) search were carried out through the climbing nudged elastic band method,[11, 12] using three images and a quick-min optimizer[13] (with very conservative line search settings such as a dynamic time step of 0.01). For selected cases, TrS searches were repeated with five images that validated the results of the three-image runs. Following Ref.[8], the force tolerance for transition state searches was 0.05 eV/Å. Given our interest in vibrational free energies, all the minima and TrS were self-evidently checked to yield positive vibrational frequency and, for the transition states, imaginary modes along the reaction coordinate.

Γ-centred harmonic vibrational modes and frequencies (ν_i) were computed by finite difference using two symmetric displacements of 0.015 Å. For each system, zero-point vibrational energies (ZPEs) and vibrational entropy contributions $-TS_{vib}$ (T: temperature, S_{vib}: vibrational entropy) were computed as:[14]

(S1) $$ZPE = \frac{1}{2}\sum_i h\nu_i$$

(S2) $$-TS_{vib} = -Tk_B \sum_i \left[\frac{h\nu_i}{k_B T}\left(e^{\frac{h\nu_i}{k_B T}} - 1\right)^{-1} - ln\left(1 - e^{-\frac{h\nu_i}{k_B T}}\right)\right]$$

with the summation running over the (3N-6) real-frequency vibrational modes (of frequency ν_i) in the system of N atoms. k_B is the Boltzmann constant. h is Planck constant. For transition states, imaginary modes were *not* considered in the calculation of ZPE and S_{vib}. Given the different initial geometries and reaction paths (Fig. 5), one and two imaginary modes are present at the HER Tafel transition states on Au(111) and Pt(111), respectively.

Given that our primary interest lies in the changes of the Tafel barrier with spin polarisation, we neglected the coverage (θ) dependent differential configurational entropy

$(-TdS_{vib}/d\theta = -Tk_B ln\left(\frac{1-\theta}{\theta}\right))$ of the adsorbed H-atoms on the metal slabs when calculating the results in Fig. 5.[10, 14] If one were to include this term in calculations where = 300 K, it would be expected to yield a systematic decrease (increase) in the initial Tafel state free energy (barrier) on both Au(111) and Pt(111) by 28.4 meV, regardless of the slab spin-polarisation.

Adsorption energies (E_{ad}) for HER Tafel initial (2H) and final (H-H)* states were calculated as:

(S3) $$E_{ad} = E_{2H/(H-H)^*} - E_{slab} - \frac{1}{2}E_{H2}$$

Where $E_{2H/(H-H)*}$ is the (free) energy of the slab with the Tafel initial/transition state, E_{slab} is the (free) energy of the bare optimised slab and E_{H2} is the energy of one H_2 molecule optimised in vacuo. As primarily interested in the free energy differences between Tafel transition and initial states (rather than absolute values of E_{ad}), no ZPE nor vibrational/translation entropy corrections[10] were added to E_{H2}. If included, these corrections would cancel out when calculating barriers based on the adsorption energy differences between Tafel initial and final state. Likewise, given the focus on the response on the slab spin-polarisation of the barrier for the evolution of one H_2 molecule from 2 H-atoms (for a surface coverage θ=0.25 ML), we did not resort to the use of differential adsorption energies for calculating fixed-coverage barriers.

Supplementary DFT results

Why (111) surfaces are studied for DFT: experimental observations

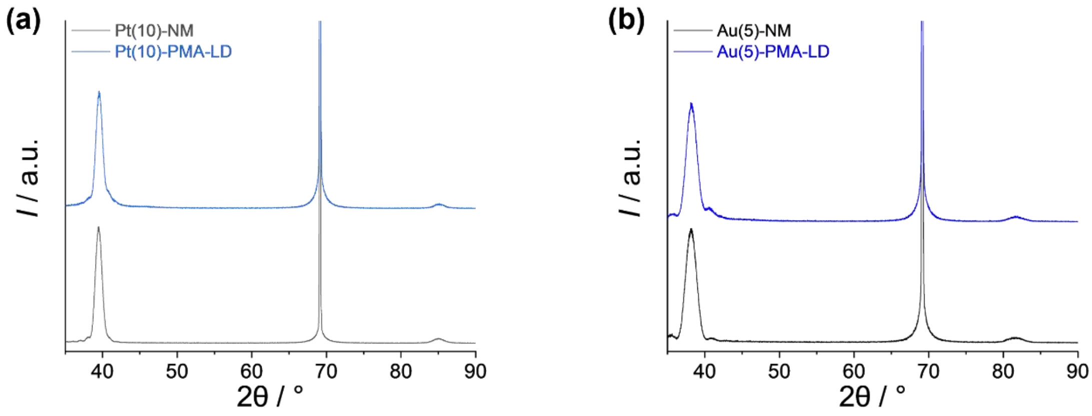

Figure S13. X-Ray Diffraction spectra of (a) Pt and (b) Au samples, showing preferential Pt(111) and Au(111) facets, respectively. For reference, Pt(111) appears at 39.5° and Au(111) at 38.2°, and their second-order diffraction peaks Pt(222) at 85.0° and Au(222) at 81.7°, with the Si substrate peak at 69.1°. The underlayer peaks are observed between 35° and 45° for α-Ta and β-Ta, and between 40° and 45° for Pt(111), Ir(111) and various facets of CoB.

Energy favoured H adsorption geometry on Pt(111): collinear vs non-collinear cases

Table S2. Relative DFT energies (eV), referenced to the lowest energy thence energy favoured system, for one H-atom adsorbed on different symmetry inequivalent geometries of the Pt(111) slab (0 μ_B/Pt-atom) with neglect (collinear DFT) and inclusion (non-collinear DFT) of spin-orbit coupling (SOC).

	TOP	FCC	HCP	Bridge
**Collinear DFT**	0.0	+0.024	+0.083	+0.065
**Non-collinear DFT**	0.0	+0.022	+0.073	+0.056

Energy favoured H adsorption geometry on Au(111): collinear vs non-collinear cases

Table S3. Relative DFT energies (eV), referenced to the lowest energy thence energy favoured system, for one H-atom adsorbed on different symmetry inequivalent geometries of the Au(111) slab (0 μ_B/Au-atom) with neglect (collinear DFT) and inclusion (non-collinear DFT) of spin-orbit coupling (SOC).

	TOP	FCC	HCP	Bridge
**Collinear DFT**	+0.172	0.0	+0.046	+0.045
**Non-collinear DFT**	+0.144	0.0	+0.044	+0.038

Table S4. Relative (collinear) DFT energies (eV), referenced to the lowest energy thence energy favoured system, for one H-atom adsorbed on different symmetry inequivalent geometries of the Au(111) slab as a function of the slab spin-polarisation (μ_B/Au-atom).

μ_B/Au-atom	TOP	FCC	HCP	Bridge
**0.0**	+0.172	0.0	+0.046	+0.045
**0.1**	+0.129	0.0	+0.041	+0.031
**0.2**	+0.165	0.0	+0.048	+0.041
**0.3**	+0.135	0.0	+0.050	+0.034
**0.4**	+0.124	0.0	+0.045	+0.030

Optimised 2H and (H-H)* geometries on Au(111) as a function of spin-polarisation

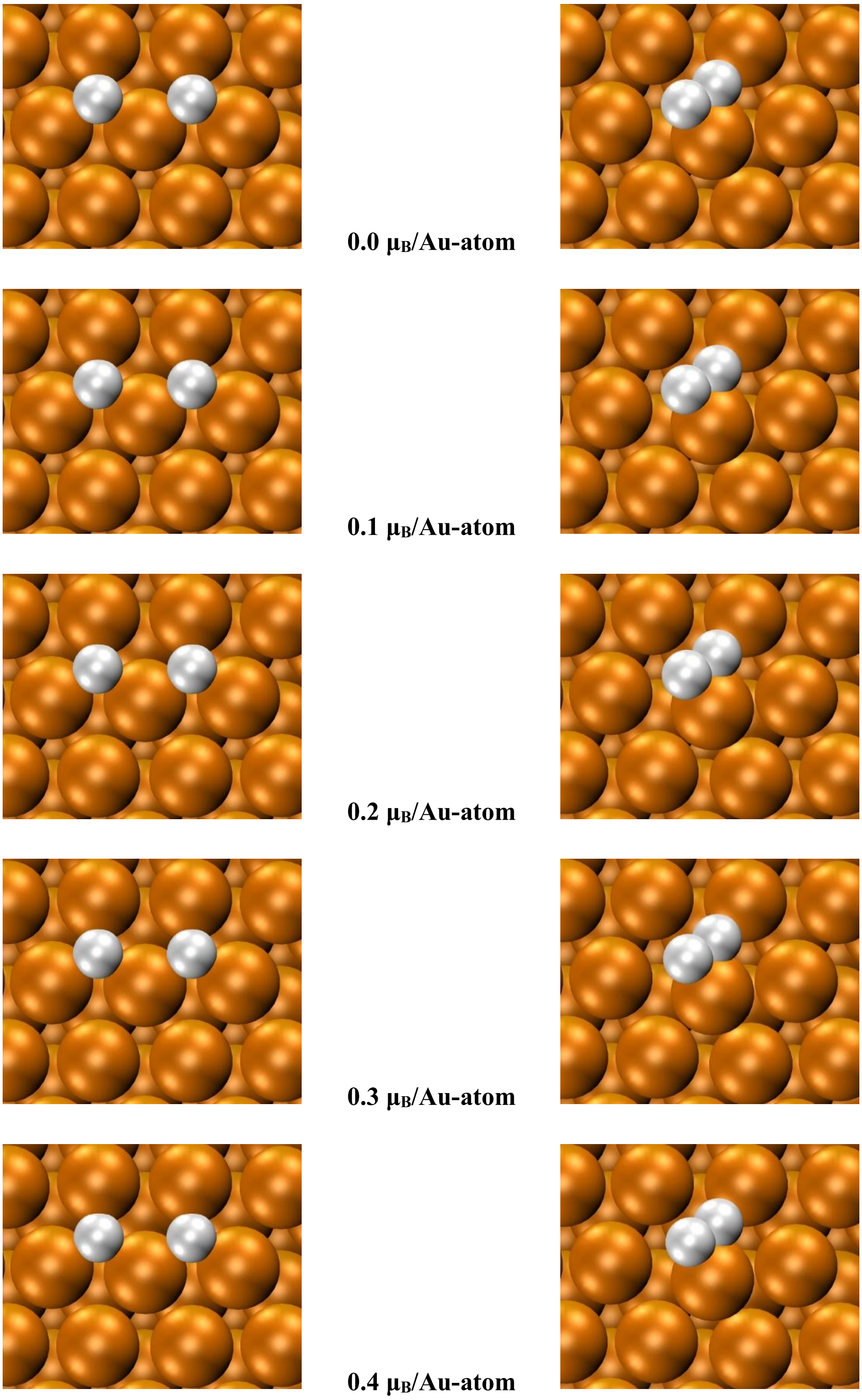

Figure S14. Optimised geometries for the initial (*left*) and transition (*right*) states of the HER Tafel reaction a function of the **Au(111)** surface spin-polarisation. Top: 0.0 μ_B/Au-atom, Bottom: 0.4 μ_B/Au-atom.

Optimised 2H and (H-H)* geometries on Pt(111) as a function of spin-polarisation

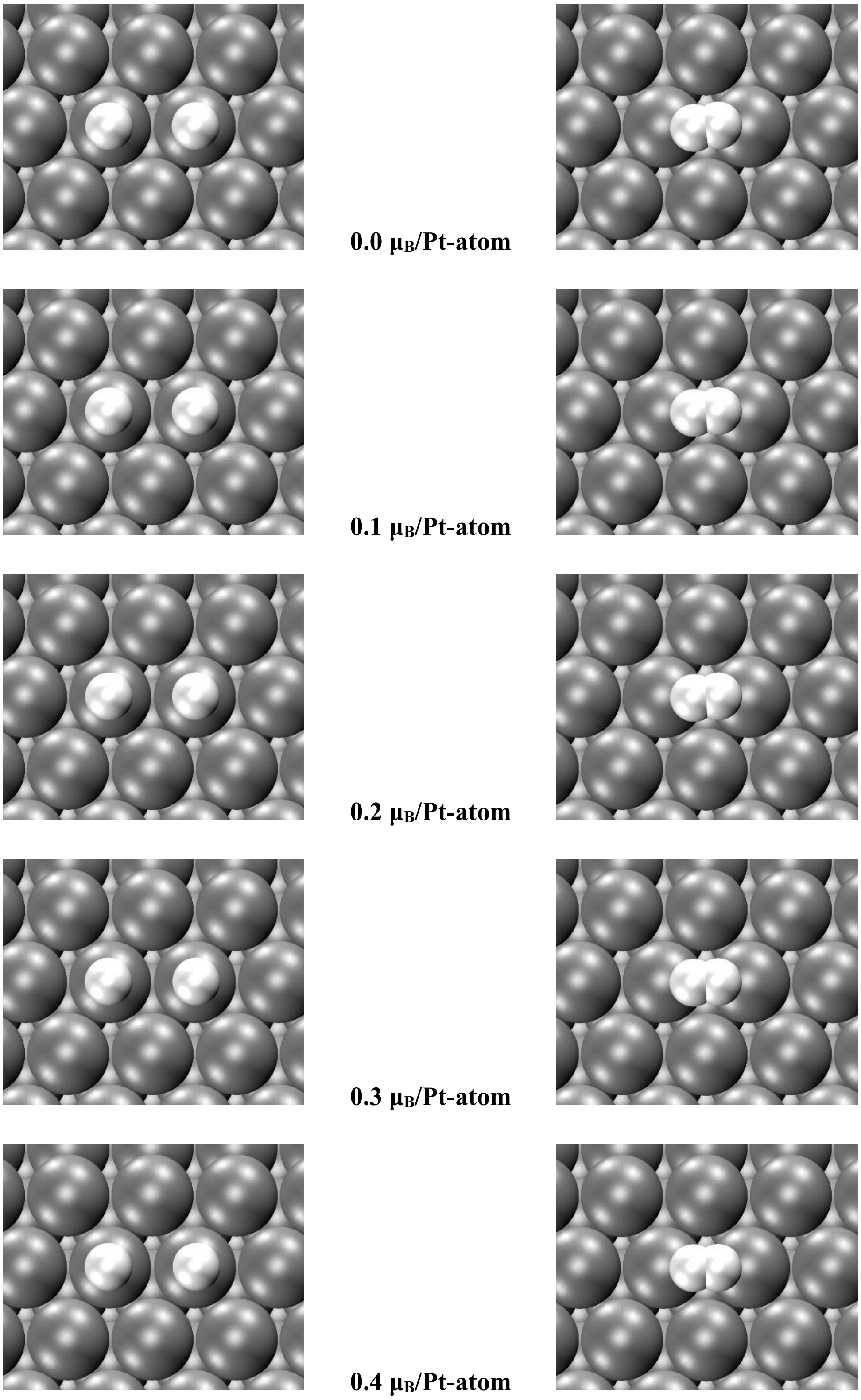

Figure S15. Optimised geometries for the initial (*left*) and transition (*right*) states of the HER Tafel reaction a function of the **Pt(111)** surface spin-polarisation. Top: 0.0 μ_B/Pt-atom, Bottom: 0.4 μ_B/Pt-atom.

Magnetic moments on the H-atoms for the HER Tafel initial and transition states

Table S5. Calculated magnetic moments on the H-atoms (H PAW core-spheres) for the HER Tafel initial (2H) and transition [(H-H)*] states as a function of the overall slab spin-polarisation (μ_B/metal-atom). The forward slash symbol ("/") is used to separate the results for each of the two H-atoms in the systems.

	**Au(111)**		**Pt(111)**	
**μ_B/metal-atom**	**2H**	**(H-H)***	**2H**	**(H-H)***
**0.0**	0.000/0.000	0.000/0.000	0.000/0.000	0.000/0.000
**0.1**	0.011/0.011	0.013/0.009	-0.001/-0.001	0.001/0.001
**0.2**	0.026/0.026	0.030/0.019	-0.001/-0.001	0.002/0.002
**0.3**	0.038/0.038	0.044/0.026	-0.001/-0.001	0.004/0.003
**0.4**	0.047/0.047	0.059/0.036	-0.001/-0.001	0.005/0.005

Role of spin-orbit coupling for the calculated HER Tafel Barriers

We numerically quantified the role of spin-orbit coupling (SOC) for the computed HER Tafel barriers on the system with expectedly larger spin-orbit coupling [Pt(111)] in the absence of spin-polarisation (0 μ_B/Pt-atom). Neglect or inclusion of SOC in the simulations leads to deviations smaller than 7 meV between collinear (no SOC) and fully self-consistent non-collinear simulations (SOC). In the absence of the underlying PMA substrate, neglected in the simulations, the non-magnetic ground state for the systems studied prevents self-consistent non-collinear transition-state search for nonzero slab spin-polarisations as the system naturally relaxes into a 0 μ_B/Pt-atom state. However, the systematically overwhelming contributions from the *s* ($l = 0$) states of the H-atoms for both the Tafel initial and transition states with the negligible *p* (*d*) rehybridisation regardless of the slab spin-polarisation suggest inevitably negligible SOC-effects on the HER Tafel barrier, which ultimately involves only *s* electrons (no orbital angular momentum as $l = 0$) in H-containing (Z=1) subsystems.

Dependence of E_{DFT}, ZPE and $-TS_{vib}$ (T=300 K) on the slab spin-polarisation

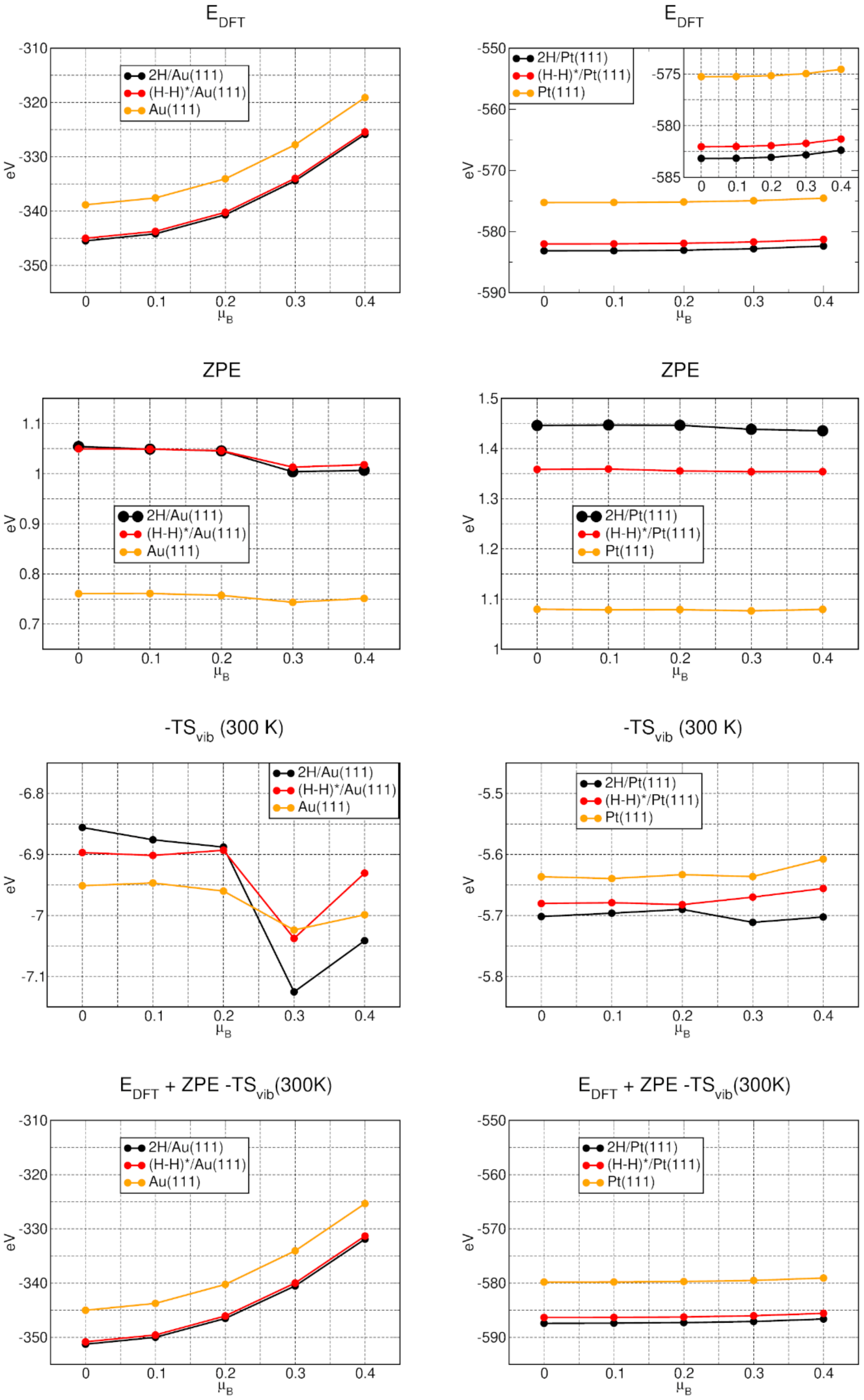

Figure S16. From top to bottom, the calculated dependence on the slab spin-polarisation (μ_B/atom) of the DFT energy (E_{DFT}), ZPE, $-TS_{vib}$ (T=300 K), and total free energy (E_{DFT} + ZPE $-TS_{vib}$) for the bare slabs, the Tafel initial state (2H), and Tafel transition state (H-H)* on Au(111) (*left*) and Pt(111) (*right*) slabs. To facilitate comparison, the same vertical-axis scale has been used for the Au(111) and Pt(111) results. The inset in the E_{DFT} picture for Pt(111) reports a close up of the data to highlight the non-linear change with μ_B.

Convergence of the ZPE and $-TS_{vib}$ contributions to the HER Tafel barrier as a function of the number of vibrationally active Au(111) layers in the slab

Figure S17 indicates that, in the absence of spin-polarisation (0 μ_B/Au-atom), the ZPE and $-TS_{vib}$ contributions to the Tafel HER barrier show a rapid convergence with respect to the number of vibrationally active Au(111) layers included in the simulations. Going from two to three Au(111) layers, the ZPE and $-TS_{vib}$ change by less than 2 meV in absolute value, for a total convergence of the overall ZPE$-TS_{vib}$ contribution to within 3.5 meV.

The same test carried out for the Au(111) slab with a spin-polarisation of 0.3 μ_B/Au-atom shows different trends. The deviations in the ZPE contribution to the HER Tafel barrier going from two to three vibrationally active Au(111) layers remain smaller than 2 meV, and are further reduced to less than 1 meV passing from three to four vibrationally active layers. In contrast, the $-TS_{vib}$ contribution shows the absence of any convergence with the number of vibrationally active layers accessible with our models (four at most, due to the use of five-layers slabs with the bottommost kept fixed during geometry optimisations). As seen in Fig. S17, the changes in $-TS_{vib}$ contribution to the barrier increase by 13.6 meV and 20.8 meV going from two to three, and from three to four vibrationally active Au(111) layers. While not absolute, this level of convergence is nevertheless over six time smaller than the calculated change in the total free- energy (E_{DFT}+ZPE$-TS_{vib}$) barrier of ~140 meV going from 0 to 0.3 μ_B/Au-atom (Fig. 5e), suggesting it as a qualitatively meaningful result at the very least.

Finally, we note that the lack of absolute convergence with the number of Au(111) layers included in the calculation for $-TS_{vib}$ (Fig. S17) results in an *under-estimation* of the $-TS_{vib}$ contribution to the barrier increase (Fig. 5g). It thus follows that the results in Fig. 5g [for three vibrationally active Au(111) layers] should be taken as a *lower boundary* for the increase of the Tafel HER barrier with the Au(111) spin-polarisation. Based on the trends in Fig. S17, in the presence of a fully converged $-TS_{vib}$ contribution, the increase in Tafel HER barrier will be larger.

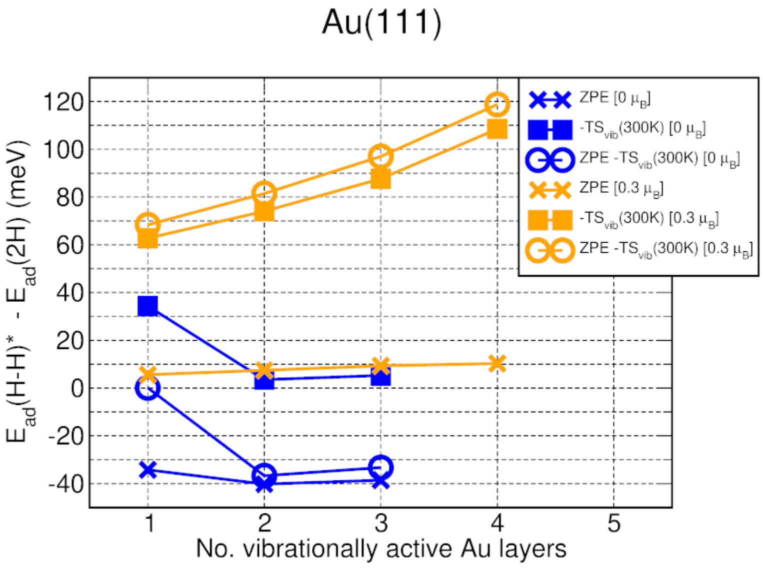

Figure S17. Converge of the ZPE and $-TS_{vib}$ contributions to the HER Tafel reaction barrier on Au(111) as a function of the number of active Au layers in the vibrational modes calculations. The units of the vertical axis are meV, and the vertical scale is the same as in Fig. 5g–h.

Dependence of E_{ad} on the slab spin-polarisation

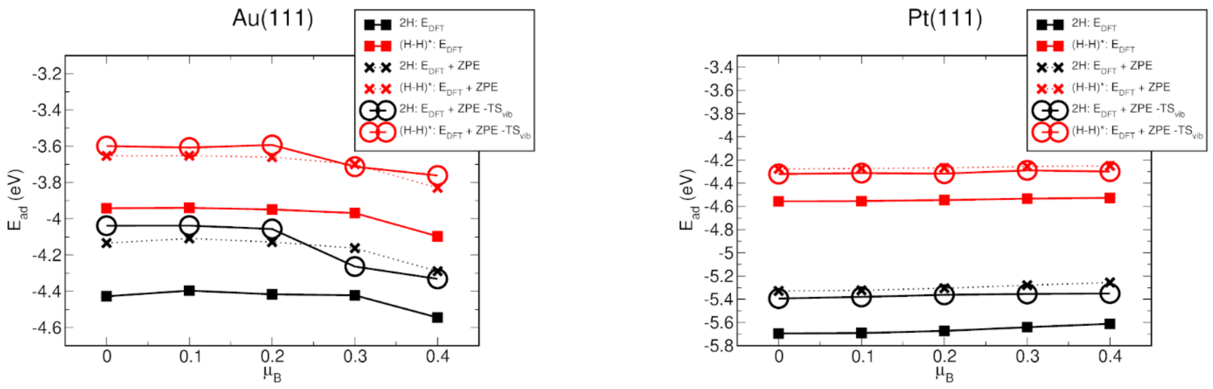

Figure S18. Calculated adsorption energy for HER Tafel initial (2H) and transition (H-H)* states on Au(111) (*left*) and Pt(111) (*right*) as a function of the use of E_{DFT}, E_{DFT}+ZPE and E_{DFT} + ZPE$-TS_{vib}$. The barriers shown in Fig. 5e–f equal the difference between 2H and (H-H) adsorption energy in this graph.

Dependence of d-band centre (ε_d) the slab spin-polarisation

The d-band centre (ε_d) was computed from the first moment of the atom and d-states resolved Density of States $n_d(E)$:

(S4) $$\varepsilon_d = \frac{\int_{-\infty}^{+\infty} n_d(E)(E-E_F)dE}{\int_{-\infty}^{+\infty}(E-E_F)dE}$$

Where E_F is the Fermi level, and a 0.1 eV Gaussian smearing was used to compute $n_d(E)$ for the three (two) Au (Pt) atoms closest to the two H-atoms. The same convention was used also for the bare Au(111) and Pt(111) slabs.

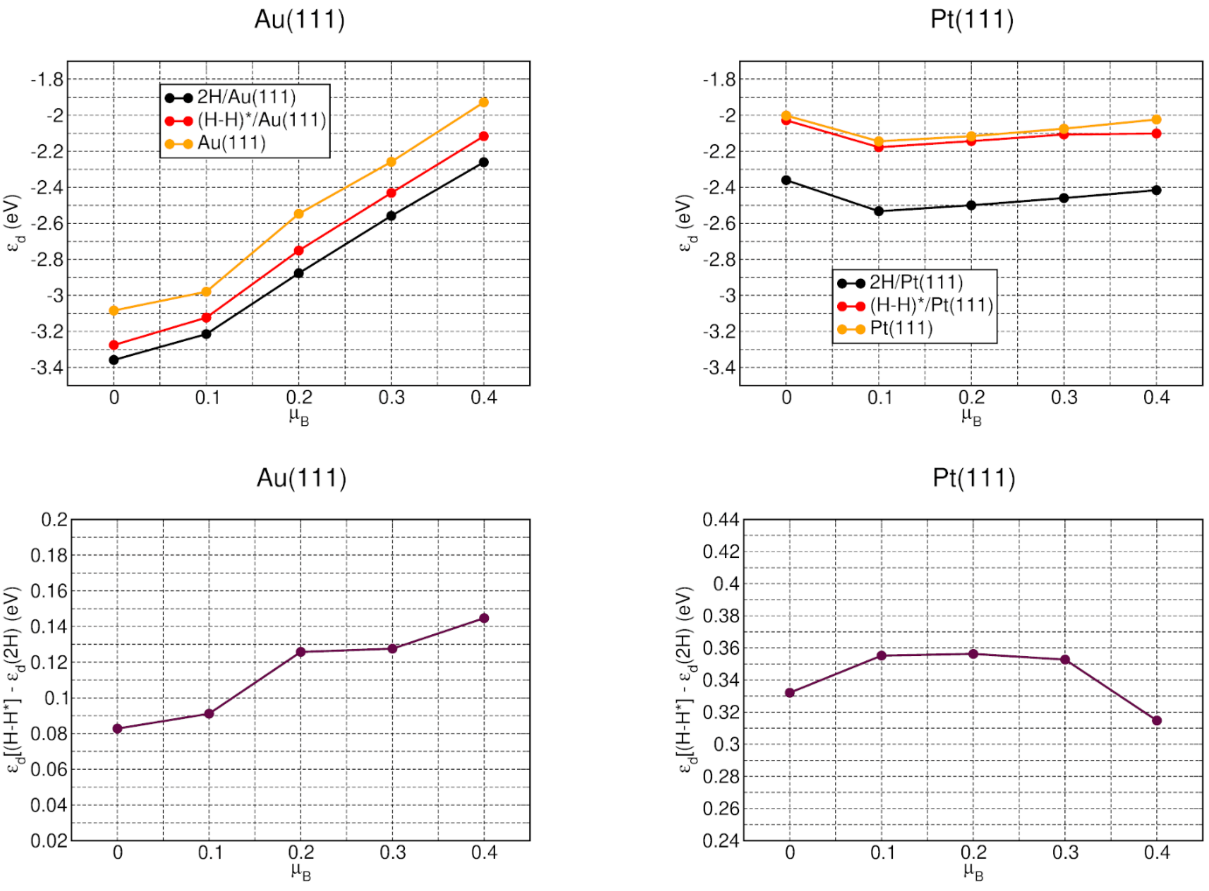

Figure S19. Dependence of the d-band centre (ε_d) on spin-polarisation for the bare slabs, the Tafel initial state (2H), and Tafel transition state (H-H)* on Au(111) (*left*) and Pt(111) (*right*) slabs. The bottom panels report the difference in ε_d between (H-H)* and 2H.

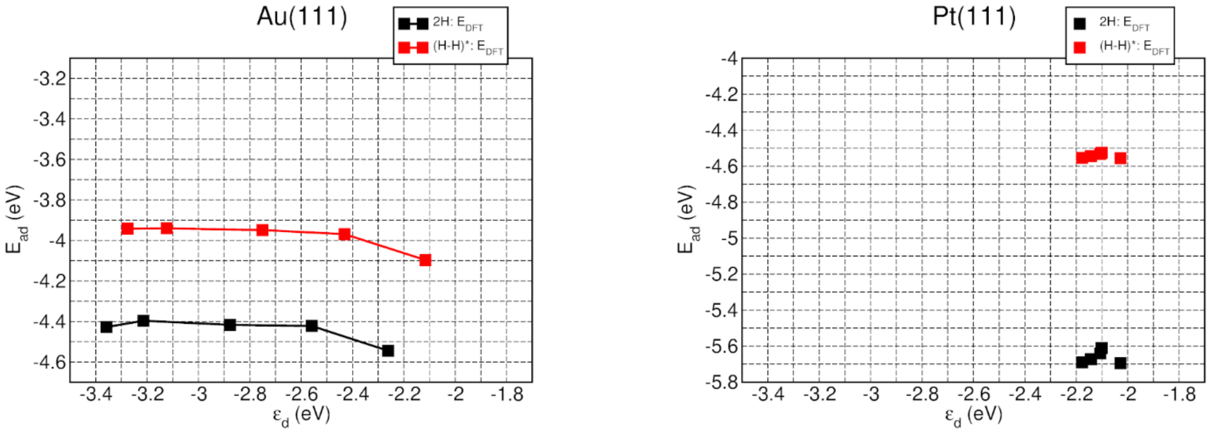

Figure S20. Dependence of the calculated DFT adsorption energy (E_{ads}, Fig. S18) on the d-band centre (ε_d, Fig. S19) due to the Au(111) (*left*) and Pt(111) (*right*) slab spin-polarisation.

Dependence of 2H and (H-H)* electronic structure on the slab spin-polarisation

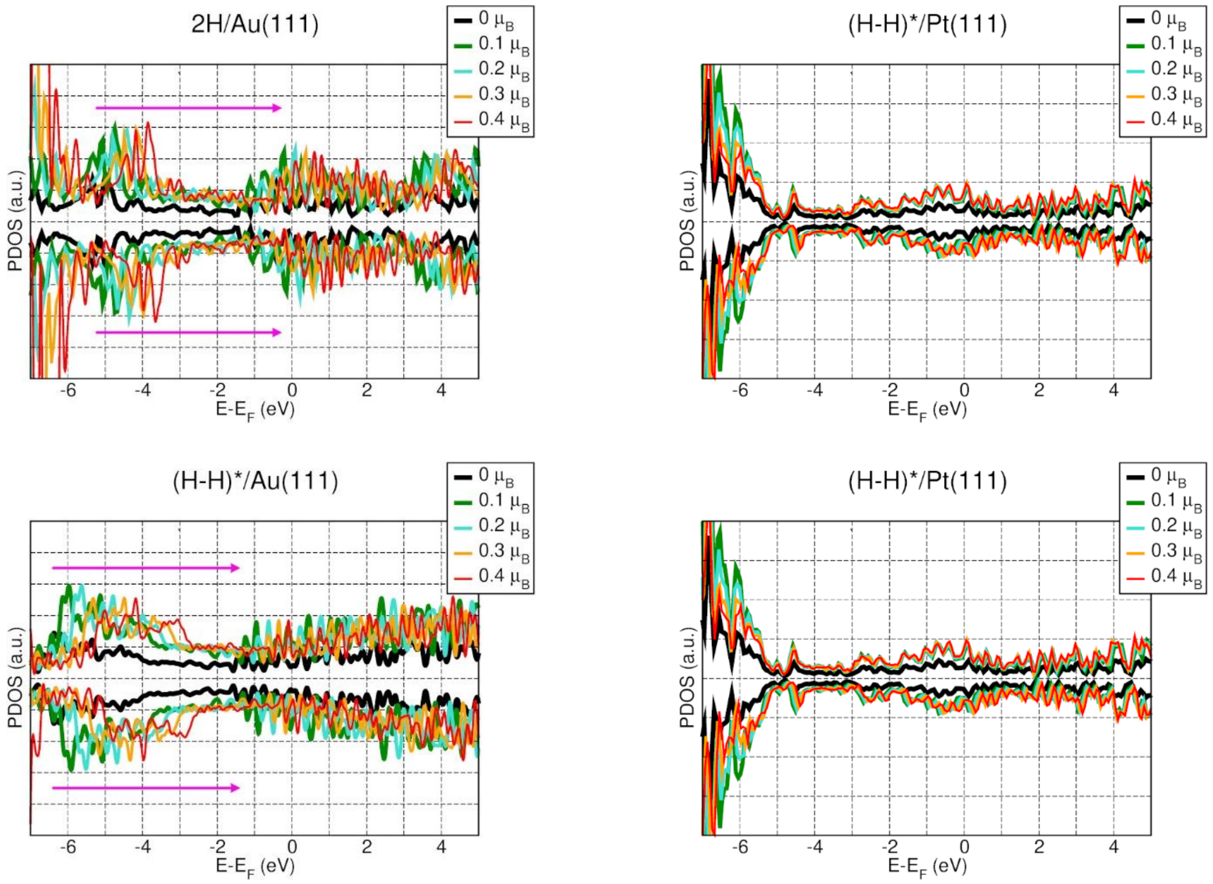

Figure S21. Calculated H-projected electronic density of states (PDOS) for the HER Tafel initial (2H, *top*) and transition [(H-H)*, *bottom*] states on the Au(111) (*left*) and Pt(111) (*right*) slabs as a function of the slab spin-polarisation [μ_B/Au(Pt)-atom]. The magenta arrows highlight the noticeable larger upward shift of the 2H and (H-H)* PDOS traces on Au(111) with spin-polarisation by comparison to the Pt(111) results.

Dependence of H-containing vibrational modes on the slab spin-polarisation

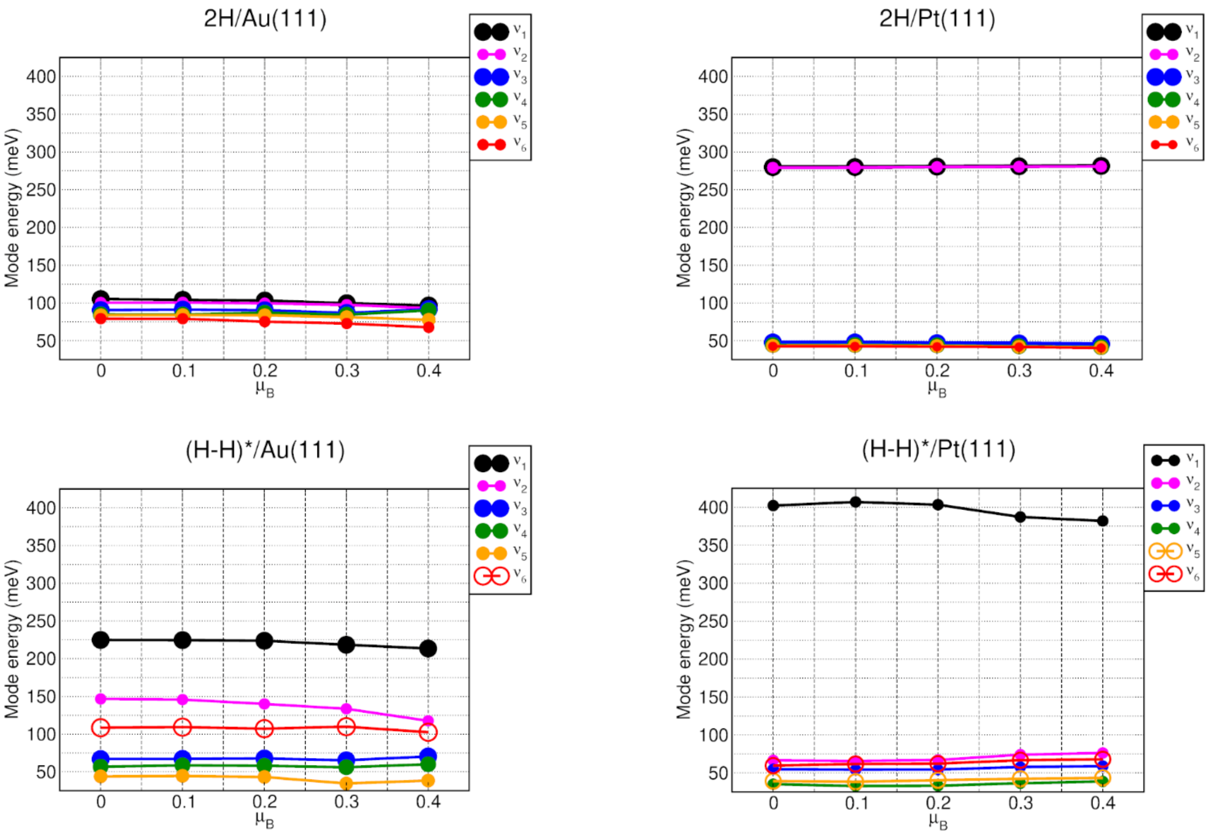

Figure S22. Calculated change in the energy of the H-dominated vibrational modes (υ_1 to υ_6) at the HER Tafel initial (2H) and transition state (H-H)* on Au(111) (*left*) and Pt(111) (*right*). υ_1 to υ_6 are the vibrational modes with the largest displacement for the H-atoms in the corresponding eigenvector. Imaginary modes at the transition states have been marked by empty circles.

Dependence of Au or Pt vibrational modes on the slab spin-polarisation

To analyse in compact fashion the role of the 2H and (H-H)* states in affecting the dependence of the Au- and Pt-dominated vibrational mode energies with the slab spin-polarisation, we recur to the associated phonon Density of States (ph-DOS) computed by smearing the calculated vibrational eigen-spectrum with Gaussian functions of standard deviation σ=0.5 meV.

As seen in Fig. S23, the band of the Au and Pt dominated modes (phonons) show both different energies and width, with the band for Au spanning a narrower ~17.5 meV window in contrast to the wider Pt one (~22.5 meV). Whereas the presence of a spin-polarisation in the 2H/Au(111) system systematically shifts the Au phonon-band to lower energies (see also Fig. S24), the dependence for the (H-H)*/Au(111) states is more complex, with an initial shift to higher energies followed by a

reduction in energies as the spin-polarisation grows larger than 0.2 μ_B. These changes are larger than seen for the bare Au(111) slab indicating a non-negligible role of the bonding of 2H and (H-H)* in altering the details of the phonon distribution for the underlying Au(111) slab. In contrast, the calculated changes in ph-DOS for 2H and (H-H)* on Pt(111) appear to be more similar, regardless of the slab spin-polarisation (Fig. S24).

As a result of these differences, on Au(111), the calculated changes for the 2H state are roughly twice those for the H-H)*. In contrast, on Pt(111) the spin-polarisation induced changes on the initial (2H) and transition [(H-H)*] states are nearly identical.

Fig. S25 extends the analysis by considering the difference in ph-DOS between the transition, (H-H)*, and initial, 2H, Tafel states on both Au(111) and Pt(111). The changes on Au(111) are more pronounced than on Pt(111) with a more complex dependence of the ph-DOS on the energy of the modes and the spin-polarisation. In addition, on Au(111), the spin-polarisation results in increase and decrease of the (low S_{vib}) high-energy and (high S_{vib}) low-energy ph-DOS shoulders of the ph-DOS. These changes in turn determine the entropy ($-TS_{vib}$) driven relative stabilisation of 2H state with respect to the (H-H)* one as a function of increasing spin-polarisation, leading to an overall increase in the Tafel barrier (Fig. 5e–g).

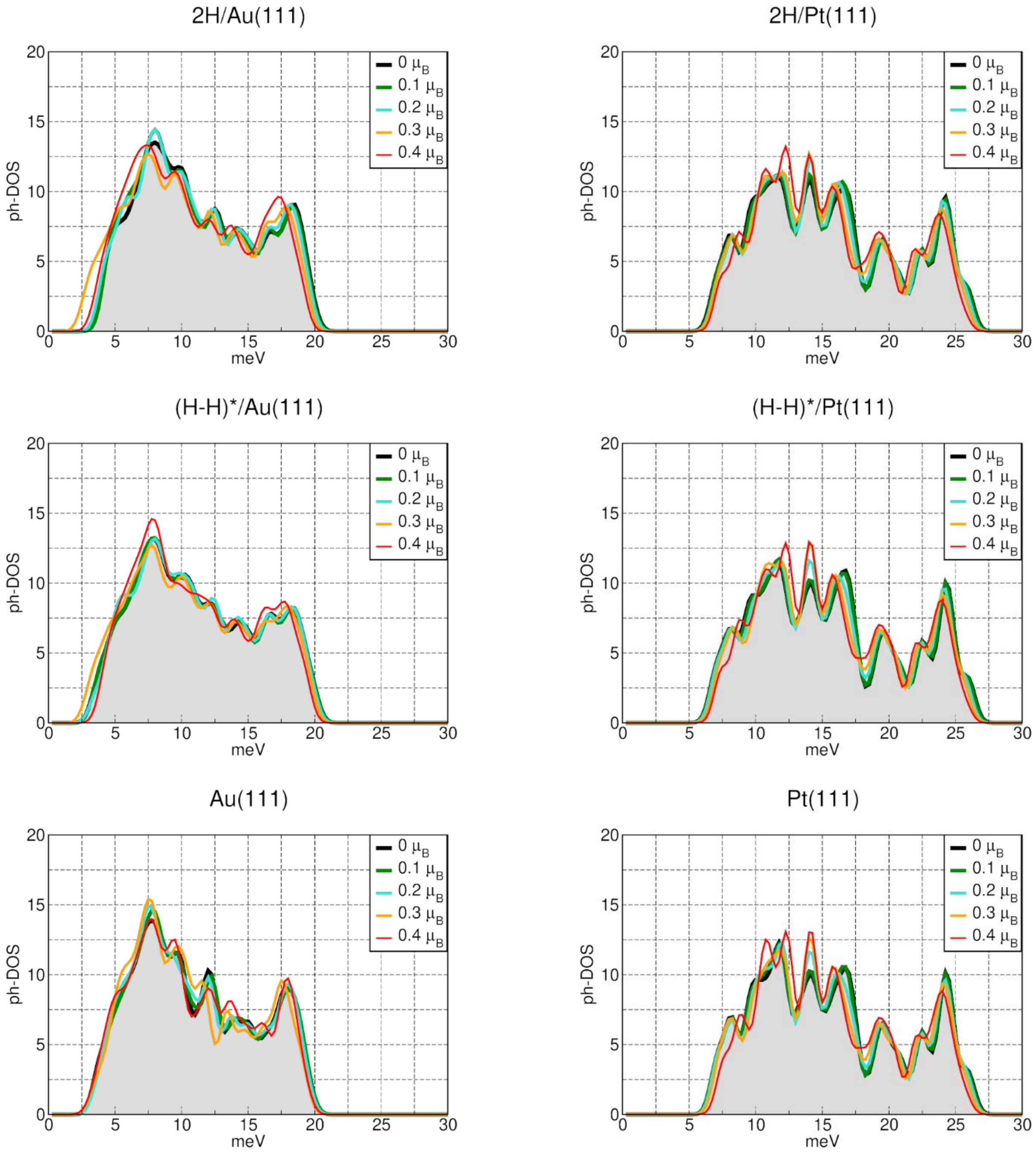

Figure S23. Calculated vibrational density of states (ph-DOS) for the Au- and Pt-dominated vibrational modes (phonons) at the HER Tafel initial (2H, *top*) and transition [(H-H)*, *middle*] states on the Au(111) (*left*) and Pt(111) (*right*) slabs as a function of the slab spin-polarisation [μ_B/Au(Pt)-atom]. Results for the bare (111) slabs are reported in the bottom panels.

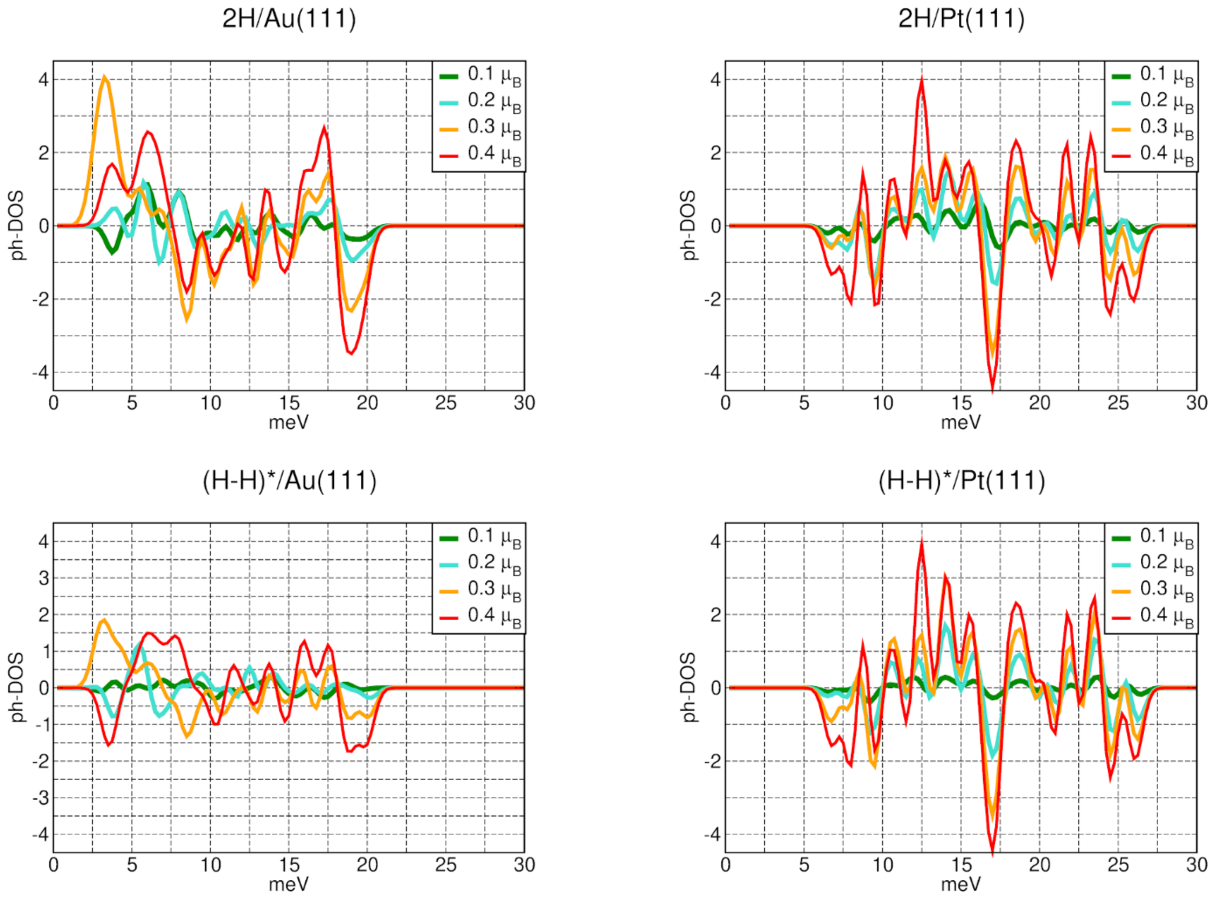

Figure S24. Spin-polarisation induced changes in the calculated vibrational density of states (ph-DOS) in Fig. S23 for the HER Tafel initial (2H, *top*) and transition [(H-H)*, *bottom*] states on the Au(111) (*left*) and Pt(111) (*right*) slabs. Each trace represents the difference in ph-DOS at a given slab polarisation (as shown in Fig. S23) with respect to the results for the pristine, non-spin-polarised case (0.0 μ_B). On Au(111), the calculated changes for the initial HER Tafel state (2H) are roughly twice those for the transition state [(H-H)*]. In contrast, on Pt(111) the spin-polarisation induced changes on the initial (2H) and transition [(H-H)*] states are nearly identical.

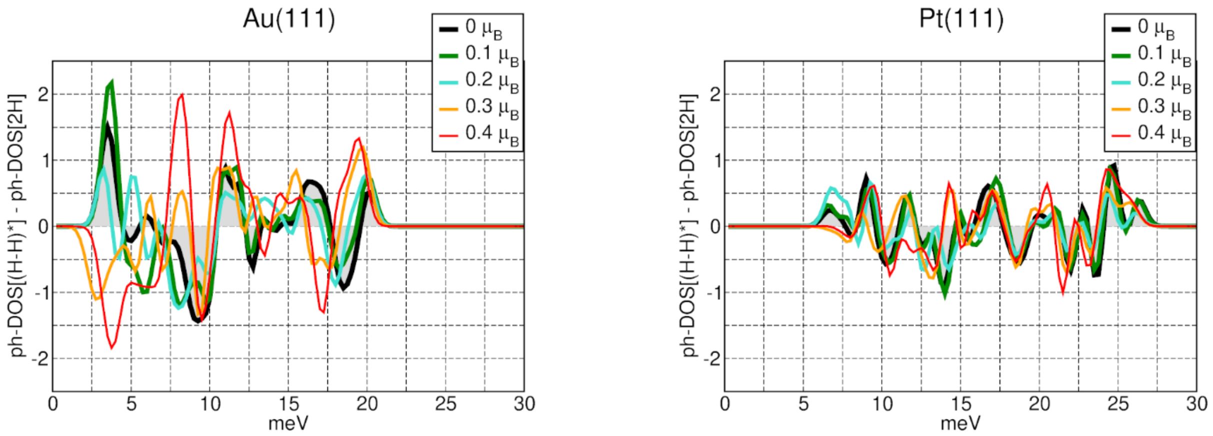

Figure S25. Difference in the calculated vibrational density of states (ph-DOS) in Fig. S24 for the HER Tafel transition [(H-H)*] and initial (2H) states on the Au(111) (*left*) and Pt(111) (*right*) slabs as a function of the slab spin-polarisation. Each trace is the difference in ph-DOS between (H-H)* and 2H (as shown in Fig. S23) at a given slab polarisation. The changes on Au(111) are more pronounced than on Pt(111) with a more complex dependence of the ph-DOS on the energy of the modes and the spin-polarisation. In addition, on Au(111), the spin-polarisation results in increase and decrease of the (low S_{vib}) high-energy and (high S_{vib}) low-energy ph-DOS shoulders of the ph-DOS. These changes in turn drive the relative stabilisation of 2H state with respect to the (H-H)* one, determining the overall increase in the Tafel barrier with the slab spin-polarisation (Fig. 5).

References:

(1) Kodaimati, M. S.; Gao, R.; Root, S. E.; Whitesides, G. M. Magnetic fields enhance mass transport during electrocatalytic reduction of CO_2. *Chem Catal.* **2022**, *2* (4), 797–815. DOI: 10.1016/j.checat.2022.01.023.

(2) Vensaus, P.; Liang, Y.; Ansermet, J.-P.; Soler-Illia, G. J. A. A.; Lingenfelder, M. Enhancement of electrocatalysis through magnetic field effects on mass transport. *Nat. Commun.* **2024**, *15* (1), 2867. DOI: 10.1038/s41467-024-46980-8.

(3) Xue, S.; Garlyyev, B.; Watzele, S.; Liang, Y.; Fichtner, J.; Pohl, M. D.; Bandarenka, A. S. Influence of Alkali Metal Cations on the Hydrogen Evolution Reaction Activity of Pt, Ir, Au, and Ag Electrodes in Alkaline Electrolytes. *ChemElectroChem* **2018**, *5* (17), 2326–2329. DOI: 10.1002/celc.201800690.

(4) Goyal, A.; Koper, M. T. M. The Interrelated Effect of Cations and Electrolyte pH on the Hydrogen Evolution Reaction on Gold Electrodes in Alkaline Media. *Angew. Chem. Int. Ed.* **2021**, *60* (24), 13452–13462. DOI: 10.1002/anie.202102803.

(5) Marcandalli, G.; Boterman, K.; Koper, M. T. M. Understanding hydrogen evolution reaction in bicarbonate buffer. *J. Catal.* **2022**, *405*, 346–354. DOI: 10.1016/j.jcat.2021.12.012.

(6) Deng, G.-H.; Zhu, Q.; Rebstock, J.; Neves-Garcia, T.; Baker, L. R. Direct observation of bicarbonate and water reduction on gold: understanding the potential dependent proton source during hydrogen evolution. *Chem. Sci.* **2023**, *14* (17), 4523–4531. DOI: 10.1039/D3SC00897E.

(7) Gisbert-González, J. M.; Rodellar, C. G.; Druce, J.; Ortega, E.; Cuenya, B. R.; Oener, S. Z. Bias Dependence of the Transition State of the Hydrogen Evolution Reaction. *J. Am. Chem. Soc.* **2025**, *147* (6), 5472–5485. DOI: 10.1021/jacs.4c18638.

(8) Lindgren, P.; Kastlunger, G.; Peterson, A. A. A Challenge to the G ∼ 0 Interpretation of Hydrogen Evolution. *ACS Catal.* **2020**, *10* (1), 121–128. DOI: 10.1021/acscatal.9b02799.

(9) van der Heijden, O.; Park, S.; Vos, R. E.; Eggebeen, J. J. J.; Koper, M. T. M. Tafel Slope Plot as a Tool to Analyze Electrocatalytic Reactions. *ACS Energy Lett.* **2024**, *9* (4), 1871–1879. DOI: 10.1021/acsenergylett.4c00266.

(10) Skúlason, E.; Karlberg, G. S.; Rossmeisl, J.; Bligaard, T.; Greeley, J.; Jónsson, H.; Nørskov, J. K. Density functional theory calculations for the hydrogen evolution reaction in an electrochemical double layer on the Pt(111) electrode. *Phys. Chem. Chem. Phys.* **2007**, *9* (25), 3241–3250. DOI: 10.1039/B700099E.

(11) Henkelman, G.; Jónsson, H. Improved tangent estimate in the nudged elastic band method for finding minimum energy paths and saddle points. *J. Chem. Phys.* **2000**, *113* (22), 9978–9985. DOI: 10.1063/1.1323224.

(12) Henkelman, G.; Uberuaga, B. P.; Jónsson, H. A climbing image nudged elastic band method for finding saddle points and minimum energy paths. *J. Chem. Phys.* **2000**, *113* (22), 9901–9904. DOI: 10.1063/1.1329672.

(13) Sheppard, D.; Terrell, R.; Henkelman, G. Optimization methods for finding minimum energy paths. *J. Chem. Phys.* **2008**, *128* (13), 134106. DOI: 10.1063/1.2841941.

(14) Skúlason, E.; Tripkovic, V.; Björketun, M. E.; Gudmundsdóttir, S.; Karlberg, G.; Rossmeisl, J.; Bligaard, T.; Jónsson, H.; Nørskov, J. K. Modeling the Electrochemical Hydrogen Oxidation and Evolution Reactions on the Basis of Density Functional Theory Calculations. *J. Phys. Chem. C* **2010**, *114* (42), 18182–18197. DOI: 10.1021/jp1048887.
